# Mesoporous silica nanoparticles in glioblastoma: smart nano-platforms for targeted therapy and precision diagnosis

**DOI:** 10.1007/s13205-025-04639-1

**Published:** 2026-01-20

**Authors:** Priyanka Hiremath, Gaurisha alias Resha Ramnath Naik, Amrita Arup Roy, Ritu Kudarha, Rachana S. P., Paniz Hedayat, Jayvadan Patel, Srinivas Mutalik, Abhishek Kumar Singh, Namdev Dhas

**Affiliations:** 1 Department of Pharmaceutics, Manipal College of Pharmaceutical Sciences, Manipal Academy of Higher Education (MAHE), Manipal, Udupi, Karnataka 576104 India; 2 Formulation Scientist, Aavis Pharmaceuticals, Hoschton, GA 30548 USA; 3 Manipal Centre for Biotherapeutics Research, Manipal Academy of Higher Education (MAHE), Manipal, Udupi, Karnataka 576104 India

**Keywords:** Mesoporous Silica Nanoparticles, Brain Tumor, Targeting, Surface-modification, Nanocomposites

## Abstract

Glioblastoma multiforme (GBM) is a highly aggressive type of brain cancer known for its rapid progression and treatment resistance, presenting significant challenges for effective management. This article examines the promising potential of mesoporous silica nanoparticles (MSNs) as a groundbreaking platform for both the treatment and diagnosis of this formidable disease. MSNs boast several advantageous properties, including a large surface area, customizable pore sizes, and excellent biocompatibility. These characteristics enable efficient encapsulation of therapeutic agents, controlled release, and targeted delivery directly to GBM cells. One of the key advantages of MSNs is their ability to be functionalized with specific targeting ligands, which enhances their specificity toward tumor cells, facilitates navigation through the blood–brain barrier (BBB), and helps address the issues of tumor heterogeneity and drug resistance. When integrated with multimodal therapies, such as chemotherapy, immunotherapy, and photodynamic therapy, MSNs can create synergistic effects that improve therapeutic outcomes while reducing adverse off-target effects. Additionally, MSNs are poised to enhance diagnostic capabilities, improving imaging techniques for the accurate detection and monitoring of GBM. This review consolidates recent advancements in MSN-based approaches, emphasizing their therapeutic and diagnostic potential while also discussing toxicity concerns and outlining future pathways for clinical application to ultimately enhance patient outcomes.

## Introduction

**Fig. 1:**
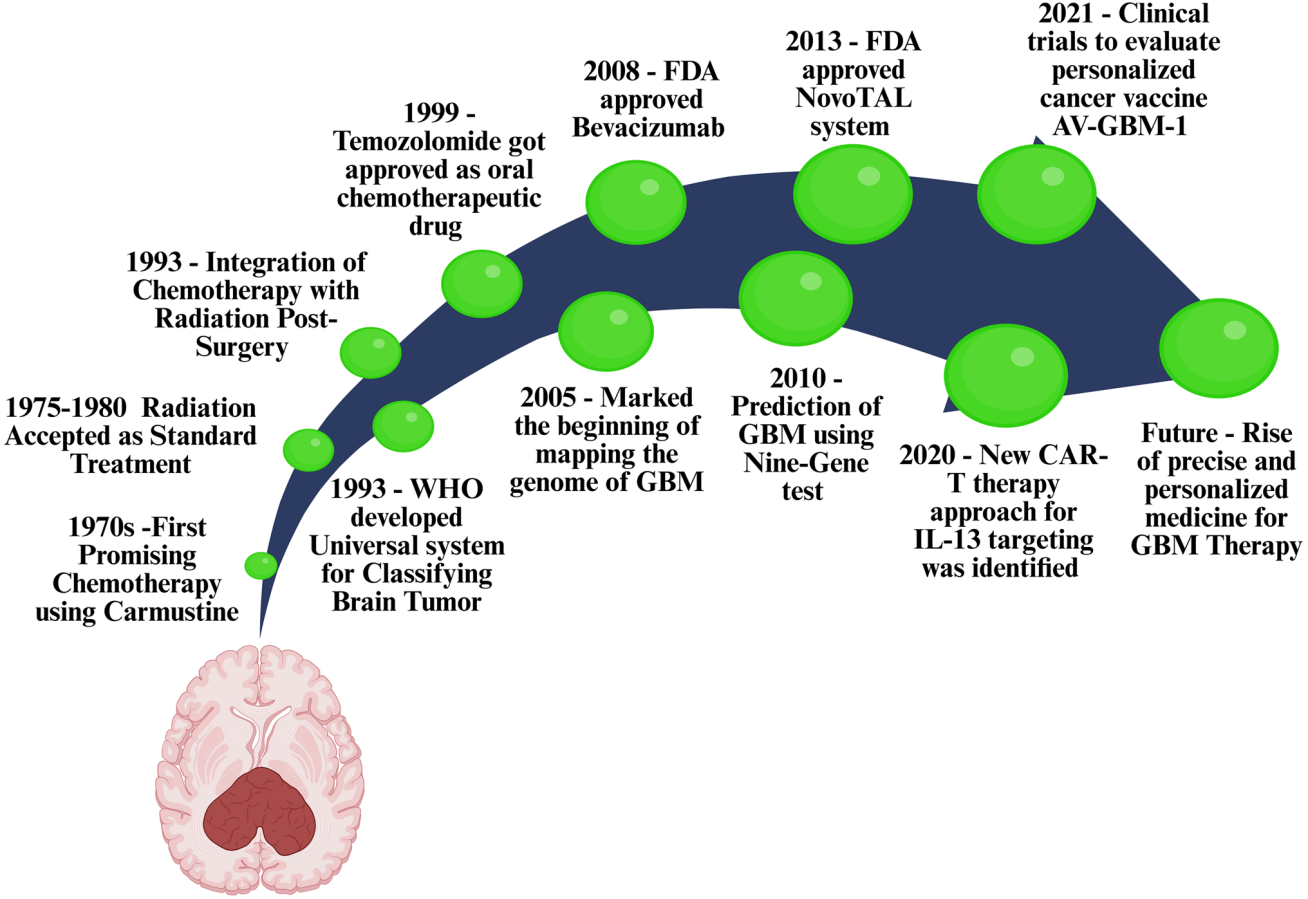
Timeline of Significant Milestones in Glioblastoma Multiforme (GBM) Therapy and Research. This timeline illustrates significant advances in GBM treatment, from its identification in the early twentieth century to modern therapies. Key milestones include surgical resection (1970s), temozolomide approval (2005), and recent nanoparticle-based therapies, highlighting progress and ongoing challenges

The American Cancer Society publishes a report on the occurrence and death rate of cancer yearly. According to the figures given, it is anticipated that in the year 2025, the United States will be afflicted by 2,041,910 new cases, resulting in 618,120 fatalities due to cancer. Among them, there will be 18,330 demises caused by brain cancer (Siegel et al. 2025). Although brain cancer is relatively rare, it is a serious condition with a high fatality rate, making it a critical health concern. Consequently, extensive research has been dedicated to this field, aiming to deepen our knowledge of cancer and develop innovative treatments to enhance outcomes for those affected (Hanif Farina et al. 2017). The brain controls and regulates all aspects of our lives as a vital part of our body and the main processing unit. Consequently, damage to any part of it may result in headaches, vision problems, hearing difficulties, seizures, and balance issues. Brain cancer is mostly a brain tumor and sometimes permeates the central nervous system (CNS) (Preusser and Marosi 2017; Tandel et al. 2019). On May 09, 2016, the World Health Organization (WHO) categorized brain tumors based on their histopathological features. As per the categorization, tumors falling under Grades I and II are deemed benign, whereas those under Grades III and IV are malignant, with the latter being generally known as glioblastoma Multiforme (GBM). GBM accounts for roughly 49% of all malignant brain tumors. It is one of the hardest-to-treat cancers, with a more than 90% recurrence rate. GBM, as the name suggests, comprises various types of glial cells, with multiform denoting a high degree of variability and diversity. This motley of GBM complicates the treatment further (Wilson et al. 2014). The major hurdle in treating CNS diseases is Blood Brain Barrier (BBB), a semi-permeable membrane which is primarily composed of microvascular endothelial cells and possesses high degree of restriction (Bors and Erdö 2019). The selective nature of the membrane and the presence of tightly connected junction complexes significantly limit the delivery of anticancer agents, posing a major challenge in treating GBM. Overcoming this barrier is crucial for effectively treating this aggressive form of cancer (Karmur et al. 2020). Another obstacle in treating GBM is the presence of small population of tumor cells resembling cancer stem cells, also known as glioblastoma stem cells (GSCs). These cells have the ability to initiate tumor growth in vivo. GSCs are highly metabolic and contribute to tumor heterogeneity. Despite numerous efforts to develop therapeutic interventions targeting GSCs, clinical trials have.

yielded disappointing results, indicating a lack of success in this area (Alves et al. 2021). As a result of GBMs, the BBB is undermined, leading to a nonhomogeneous vasculature with uneven permeability (Cui et al. 2022). This is usually known as the blood-tumor barrier (BTB). It is difficult for effective chemotherapeutic agents to target these metastatic tumors due to a combination of BBB and BTB. As a result, many methodologies have been endorsed and analyzed to combat these malignant tumors and overcome these barriers (Shergalis et al. 2018; Huang et al. 2021). The available drug therapies for glioblastoma are still few and far between. Temozolomide (TMZ) is currently the most preferred FDA-approved drug for treating this tumor(Jiapaer et al. 2018). The historical evolution of GBM therapy is illustrated in Fig. 1, which outlines key milestones in the treatment and classification of GBM. This timeline underscores how therapeutic strategies have progressed in parallel with our growing understanding of GBM biology and the emergence of precision medicine. Researchers have designed innumerable nanomedicine schemes for effective drug delivery to ensure the success of pharmacological treatment. Numerous biocompatible nano-drug delivery vehicles have caught the attention of cancer researchers. These carriers include silica nanoparticles (NPs), nanoliposomes, magnetic NPs, dendrimers, nano-micelle, gold NPs, polymersomes, and various others. Nevertheless, there are specific requirements for NPs for effective drug delivery (Cui et al. 2022). Different varieties of organic and inorganic NPs have been studied as delivery systems to meet these needs.

Mesoporous silica nanoparticles (MSNs) offer distinct advantages over other nanocarriers in GBM therapy, making them a platform with high potential for targeted drug delivery and therapy. The primary benefit of MSNs is their large surface area combined with tunable pore size and volume, which enables high loading capacity of various therapeutic agents, comprising poorly soluble drugs, genes, and photothermal agents (Khalid et al. 2020). This structural feature enables controlled as well as sustained release of drugs specifically within tumor microenvironment (TME), improving therapeutic efficiency while reducing systemic adverse effects (Chen et al. 2024). Moreover, MSNs exhibit excellent biocompatibility and chemical stability, which decreases the chances of premature degradation or immune reactions compared to some organic nanocarriers. Their inorganic silica framework provides robust structural integrity under physiological conditions, ensuring that the drug payload remains protected until it reaches the target site (Mehmood et al. 2017). Another significant advantage of MSNs is the ease of surface modification with various targeting ligands, which improves selective binding to overexpressed receptors on GBM cells, thus enhancing tumor specificity and cellular uptake (Allami et al. 2023). This targeted delivery capability is crucial for overcoming the BBB and the BTB, which are major obstacles in delivering therapeutic agents to brain tumors. Moreover, MSNs can be engineered to carry multiple therapeutic agents simultaneously, facilitating combination therapies such as chemotherapy, immunotherapy, gene therapy, photothermal, and photodynamic therapy (Hu et al. 2023). This multifunctionality addresses the heterogeneity and complexity of GBM, potentially overcoming drug resistance and improving treatment outcomes. MSNs enhance the sensitization of GBM cells to conventional therapies, such as chemotherapy and radiotherapy, by utilizing their unique properties, large surface area, tunable pore size, and surface functionalization, to improve targeted drug delivery, bypass resistance mechanisms, and increase drug accumulation within tumor cells. (Allami et al. 2023). The tunable size and surface properties of MSNs, such as optimized diameters and functionalization with targeting ligands or hydrophilic coatings, enhance their ability to cross the BBB via receptor-mediated transcytosis (if the size is below 100 nm) or improved biodistribution, which is critical factor for delivering therapeutic agents to GBM cells and improving treatment efficacy. Taken together, the unique characteristics of MSNs, such as high drug loading capacity, excellent biocompatibility, surface modifiability for targeted delivery, multifunctional therapeutic potential, and enhanced BBB penetration, make them highly promising nanocarriers for glioblastoma therapy, offering distinct advantages over other delivery systems such as liposomes and polymeric nanoparticles. The primary aim of this review is to conduct a comprehensive analysis of the potential of MSNs in treating GBM and provide updated literature. The article will focus exclusively on this subject, encompassing the latest research and advancements in the field. By delving into the unique attributes of MSNs and their potential advantages, our goal is to shed light on the promise of these nanoparticles in addressing this complex disease through this review.

## Pathophysiology of GBM

As its name suggests, GBM is a glioma that develops from the brain’s glial cells. The major types of glial cells are astrocytes, oligodendrocytes, microglia, ependymal cells, and radial glia (Guttenplan and Liddelow 2019). Glial cells are paramount to the proper functioning of the CNS, serving as its vital core by providing vital nutrients, oxygen, and stromal support (Ludwig and Das 2022). Recent research has revealed that glial cells, which were previously thought to have a subordinate role in neurotransmission, hold a significant position in it. Recent studies indicate that the emergence of new glial cells may be critically involved in developing GBMs, suggesting a primary mechanism for tumor development. Furthermore, the progression to secondary GBMs appears to be associated with the accelerated proliferation of lower-grade gliomas. This revelation opens new possibilities for understanding the pathophysiology of GBMs, offering significant promise for future research directions. As aforementioned, brain tumors are categorized primarily based on their pathological characteristics by the World Health Organization (WHO). In most cases, cause of glioblastoma is unknown. Glioblastoma has the most genetic alterations of any tumor, typically brought on by the accumulation of several mutations (Ohgaki and Kleihues 2007).

To enhance our knowledge of these malignancies regarding determining prognosis, survival time, and response to treatment, The Cancer Genome Atlas Program (TCGA) has classified GBM into four types based on molecular markers: classical, mesenchymal, neural, along with procedural, with the latest research eradicating the neural type as it was affirmed to be normal neural lineage contamination (Testa et al. 2018). The Cancer Genome Atlas oversees the TCGA project, which aims to catalog all DNA alterations that occur in various types of cancer. There are several molecular mutations that have been detected in primary and secondary GBM, with their frequency and severity varying. These mutations include LOH 10q, EGFRLOH 10q, TP53, PTEN, IDH, and MGMT promoter methylation (Ohgaki and Kleihues 2013). When EGFR is mutated in glioblastoma, it triggers a series of events that contribute to tumor growth, including proliferation, angiogenesis, differentiation, and cell survival. Chemotherapy and radiation may not be completely effective against these cancer cells and might lead to the recurrence of tumors (Taylor et al. 2019). GBM is an extensively vascularized tumor characterized by microvascular proliferation and endothelial cell hypertrophy. There is an intrinsic connection between brain tumor progression and the formation of new vessels. Many angiogenic aspects contribute to enhancing angiogenesis in brain tumors, some of which also promote normal angiogenesis (Delgado-Martín and Medina 2020).

Angiogenesis is mediated by angiopoietins, VEGF, HGF, PDGF, and MMPs. This type of cancer is characterized by a significant presence of vascular endothelial growth factor (VEGF), a critical factor involved in stimulation of angiogenesis. (Wu et al. 2021). Glioblastoma’s blood supply is marked by inconsistencies in vessel diameter and permeability, resulting in hypoxia and a lack of oxygen. It is the tumor microenvironment, along with the VEGF, that perpetuates neovascularization and tumor growth. The other angiogenic factors orchestrate the endothelial membrane receptors in GBM, which stimulate a multitude of signaling cascades that result in the proliferation, migration, and differentiation of cells needed to form new blood vessels and matrix formation (Keunen et al. 2011).

**Fig. 2:**
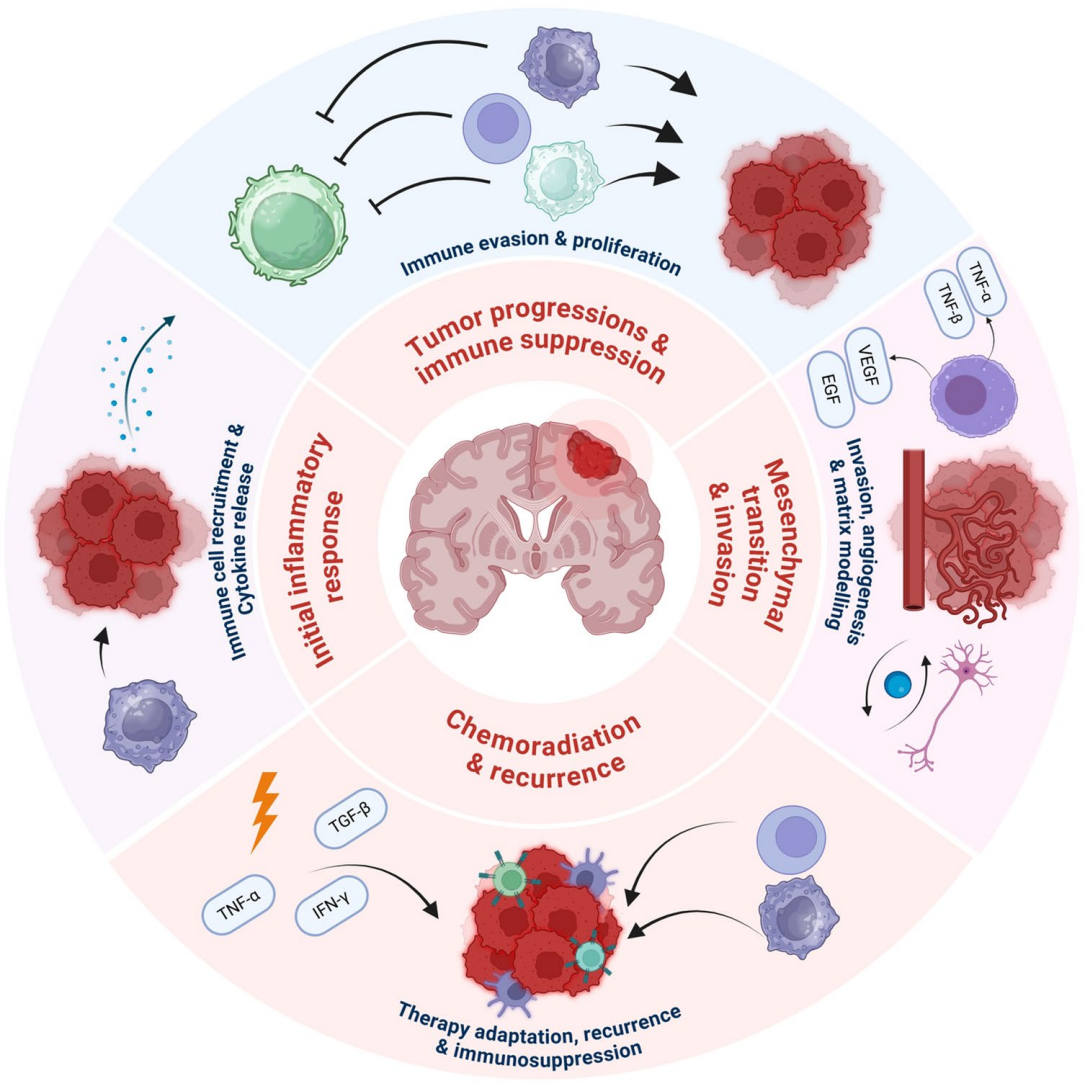
Schematic Representation of Key Pathophysiological Mechanisms in Glioblastoma Multiforme (GBM). This figure depicts core GBM mechanisms, including tumor cell proliferation, angiogenesis (via VEGF), tissue invasion, and immune evasion. It highlights genetic alterations (e.g., EGFR amplification) and signaling pathways (e.g., PI3K/AKT) driving the aggressive nature of GBM

A recent study found that ephrin receptors, a type of protein that controls cell behavior, are overly expressed in GBM and are associated with tumor growth and spread. These proteins are important in processes such as cell movement, growth, and blood vessel formation, which makes them a potential target for GBM therapy. However, their intricate signaling pathways and interactions with other proteins and molecules in the tumor environment pose significant challenges for developing effective treatments (Day et al. 2014). At the molecular level, GBM is characterized by dysregulated signaling pathways, including the phosphatidylinositol 3-kinase (PI3K)/AKT/mTOR pathway, the RAS/RAF/MAPK pathway, and the NOTCH pathway(Levine et al. 2006). The growth, movement, and survival of cells rely on these pathways. Concerning GBM, the disruption of these pathways results in uncontrolled cell growth, invasion into adjacent cerebral tissue, and the formation of new blood vessel networks to nourish the expanding tumor. This atypical cell behavior is a defining characteristic of this formidable disease (Zhang et al. 2018b). In GBM, epigenetic alterations such as DNA methylation and histone modification can influence gene expression and promote tumor growth (Nagarajan and Costello 2009). GBM cells release cytokines and chemokines that promote tumor growth and change the behavior of immune cells such as T cells, macrophages, and natural killer cells. This results in an immune system response that unintentionally supports tumor growth rather than inhibiting it. Additionally, GBM cells express molecules that can suppress the immune response (Schiffer et al. 2019). One such molecule is PD-L1, which can bind to receptors on T cells and prevent their activation and proliferation. This allows GBM cells to avoid the immune system and prevent an effective anti-tumor response. In summary, GBM cells can use several mechanisms to create an immunosuppressive microenvironment that allows them to evade the immune system and promote tumor growth (Scheffel et al. 2021; Maghrouni et al. 2021). This complex interplay of immune suppression, therapy resistance, mesenchymal transition, and tumor recurrence is visually summarized in Fig. 2**.** The diagram illustrates the central role of immune evasion, cytokine signaling, angiogenesis, and treatment adaptation in the pathophysiology of GBM.

Interleukin 13 receptor alpha 2 (IL13Rα2) is a transmembrane receptor that is upregulated in GBM cells, making it a potential target for therapy. IL13Rα2 is critical in GBM progression by activating different intracellular signaling pathways, leading to increased cell proliferation, invasion, and angiogenesis (Kahlon et al. 2004). IL13Rα2-targeted therapies have been developed to exploit its high expression in GBM cells. These therapies include immunotoxins, immune cytokines, and chimeric antigen receptor (CAR) T cells. Immunotoxins and immune cytokines are antibody-based therapies that deliver cytotoxic agents or cytokines, respectively, to IL13Rα2-expressing cells. CAR T cells are genetically engineered immune cells that express a chimeric receptor that targets IL13Rα2 on GBM cells and can potentially eliminate them. A multitude of both preclinical and clinical studies have been performed to evaluate the safety and effectiveness of IL13Rα2-targeted therapies in GBM (Kahlon et al. 2004; Thaci et al. 2014).

The results of research trials so far have been encouraging, with some patients reporting a reduction in their tumor size or a longer lifespan. It is vital to conduct more research on these treatments to improve them and solve challenges such as tumor heterogeneity and drug resistance(Liang et al. 2022). The progression of GBM is significantly influenced by the cell surface receptor, insulin-like growth factor 1 receptor (IGF-1R). In cases of GBM, it is frequently detected that the IGF-1R signaling pathway is compromised, which is associated with the accelerated growth and spreading of malignant cells, as well as a heightened ability to withstand chemotherapy and radiation treatments. In many GBM tumors, there is a lot of IGF-1R present. This means that IGF-1R could be a good target for treatment (Wang et al. 2022b). Numerous studies have demonstrated that impeding IGF-1R by inhibitors or monoclonal antibodies curtails the expansion and invasion of tumor cells, augments apoptosis, and heightens the vulnerability of GBM cells to chemotherapy and radiation. Nonetheless, additional investigation is necessary to appraise the feasibility of harnessing IGF-1R as an effective target for treating GBM (Tian et al. 2020; Wang et al. 2022b).

GBM is a highly complex and multifactorial disease that is characterized by a plethora of genetic alterations, dysregulated signaling pathways, epigenetic modifications, and an immunosuppressive TME (Nagarajan and Costello 2009; Paolillo et al. 2018). Researchers have been delving into the implementation of targeting moieties with a view to amplifying the effectiveness and precision of therapeutic agents when dealing with GBM. Targeting agents are made up of tiny molecules, peptides, antibodies, or fragments of antibodies that can specifically attach themselves to receptors located on the outer layer of GBM cells (Marei 2022). A variety of targeting moieties have been studied for the receptors present in GBM. For example, antibodies like bevacizumab have been employed to target VEGFR and hinder angiogenesis in GBM. Peptides like chlorotoxin have been found to bind specifically to matrix metalloproteinase-2 (MMP-2) and integrins, which are overexpressed on the surface of GBM cells (Rodà et al. 2023). To obtain a thorough knowledge of the capabilities of targeting moieties in treating GBM, additional research is imperative. However, initial results are promising and offer hope for the future of GBM treatment. Comprehending the underlying molecular mechanisms that govern the development and progression of GBM is of paramount importance in devising efficacious targeted therapies that can potentially ameliorate the prognosis and survival of GBM patients.

## Strategies for targeting therapeutic agents in GBM

Considering that GBM has such a high mortality rate and subpar treatment, it is imperative to find effective and novel ways to manage it. In general, treating GBM involves a diverse range of interventions, which include removing the tumor through surgery, administering chemotherapy to eradicate any residual cancerous cells, and subjecting the affected area to radiation therapy to curb the potential reoccurrence of the tumor (Fernandes et al. 2017; Yeini et al. 2021). Thus, to overcome BBB & BTB roadblocks in glioblastoma, efficient drug delivery methods are essential. TMZ is a first-line chemotherapeutic agent widely known for its cytotoxic ability to eliminate GBM tumors, as stated in numerous new studies and publications (Fernandes et al. 2017). Cytotoxicity is caused by TMZ’s ability to reposition methyl groups from adenine and guanine base pairs, leaving toxic compounds in the cell, and resulting in cell death. In some instances, MGMT (O6-methylguanine-DNA methyl transferase), which can repair cytotoxic lethal base pairs, is implicated in the emergence of resistance resulting from elevated levels (Wesolowski et al. 2010; Lee 2016). As a result of further research, it was concluded that DNA repair mechanisms and other mechanisms, such as autophagy and GSCs, contribute to TMZ resistance. For maximizing therapeutic efficacy, articulating the TMZ resistance mechanism can serve as a stable platform for specific targets (Jiapaer et al. 2018). Considering this, nanocarrier delivery has become a promising strategy, especially for drugs that need to cross the BBB. The method of preparing nanocarriers determines whether they are nano capsules, NPs, or nanospheres. When it comes to dealing with GBMs, colloidal drug carriers in the form of NPs are the go-to option. These NPs come in various forms like dendrimers, solid lipid NPs, polymeric micelles, liposomes, polymeric NPs, and silica (Hsu et al. 2021).

With a 20-year gap since the first FDA approval of the NP-based cancer treatment, there are currently numerous ongoing clinical trials, including several focused on GBM. The structure and configuration of the drug vehicle system, which is responsible for the targeting along with the delivery of therapeutics, influence the way drugs are delivered to the tumor and their resulting effectiveness (Pucci et al. 2019). Certain delivery methods employ passive or active targeting techniques to facilitate drug delivery. For example, active and passive nanoparticle targeting strategies can be used to directly target receptors on glioma tumor cells (Luiz et al. 2021).

### Passive targeting

Passive targeting can be considered a "set it and forget it" approach, as the NPs do not require any specific targeting moieties or ligands to bind to GBM cells or tissues (Hsu et al. 2021). GBM displays hypoxic regions where tumor cells are highly proliferative and mobile. As a result, targeting these areas with nanoparticle-based drug delivery or other therapeutic interventions can be challenging(Hsu et al. 2021; Barzegar Behrooz et al. 2022). Moreover, the optimal approach to exploiting the EPR effect may vary depending upon the specific characteristics of the tumor. To enhance the effectiveness of therapies, it may be imperative to employ tactics such as regulating tumor vasculature, augmenting vascular permeability, or facilitating access through the BBB. These strategies could prove crucial in improving treatment outcomes(Trivedi et al. 2023).

In GBM, the immune system’s interaction with the tumor can have an impact on its growth, spread, and response to treatment. TAMs, or Tumor-assisted microglia/macrophages, are immune cells that make up a significant portion of the GBM mass. GBM cells produce chemical signals that attract TAMs to the tumor, where they differentiate into macrophages and microglia. These TAMs are indispensable and pivotal in the progression of GBM, and high densities of TAMs have been linked to poor outcomes and higher cancer grades (Andersen et al. 2022; Wang et al. 2022a). The chemicals produced by GBM cells encourage TAMs to develop an immunosuppressive and tumor-supportive phenotype (TAM2) rather than an immunostimulatory and anti-tumor phenotype (TAM1). TAM2 promotes angiogenesis and tissue remodeling, which drives the progression of GBM. Furthermore, TAMs secrete pro-angiogenic substances like VEGF, contributing to the development of hypervascularity in GBM. It is of great magnitude to state that TAMs have a major impact on the promotion of tumor growth and advancement, as they assume an immunosuppressive as well as tumor-promoting characteristic. This phenomenon is critical to consider when examining the mechanisms behind tumor development and progression (Mantovani et al. 2017).

In GBM, the blood vessels become more "leaky," which allows immune cells and drugs to enter the tumor more easily. However, the way the tumor is structured can also affect how drugs move through it. TAMs can swallow and get rid of foreign materials, including drugs. As a result, many drugs that enter the tumor end up in these TAMs. This can be useful for accumulating drugs in the tumor and treating it. Therefore, researchers are investigating ways to alter the polarization of TAMs towards an anti-tumor phenotype by either depleting TAMs or modifying their phenotype to be more immunostimulatory(Kuntzel and Bagnard 2022). It means that changing the behavior and function of TAMs by manipulating their polarization is being explored as a potential new strategy to treat GBM(Van Dalen et al. 2018). By transforming the TAMs from a pro-tumor M2 phenotype to an opposing M1 phenotype, there exists a chance to decelerate or impede the progression of the tumor (Feng et al. 2019; Martin et al. 2020).

### Active targeting

Active targeting can be employed as an alternative when passive targeting of NPs cannot transport them across the BBB. It involves altering the composition of NPs in such a way that enables them to bind specifically to receptors on the outer surface of brain cells (Bors and Erdö 2019; Jena et al. 2020). The modification made to the composition of these minute particles eases their traversal through the blood–brain barrier, thus amplifying their effectiveness in transporting drug molecules to the brain. Such targeted delivery mechanisms hold immense promise for the treatment of neurological disorders. By focusing on a specific approach, it is anticipated that he administration of drugs to the brain will be amplified, which could potentially lead to better results in the treatment process(Puris et al. 2022).

**Fig. 3:**
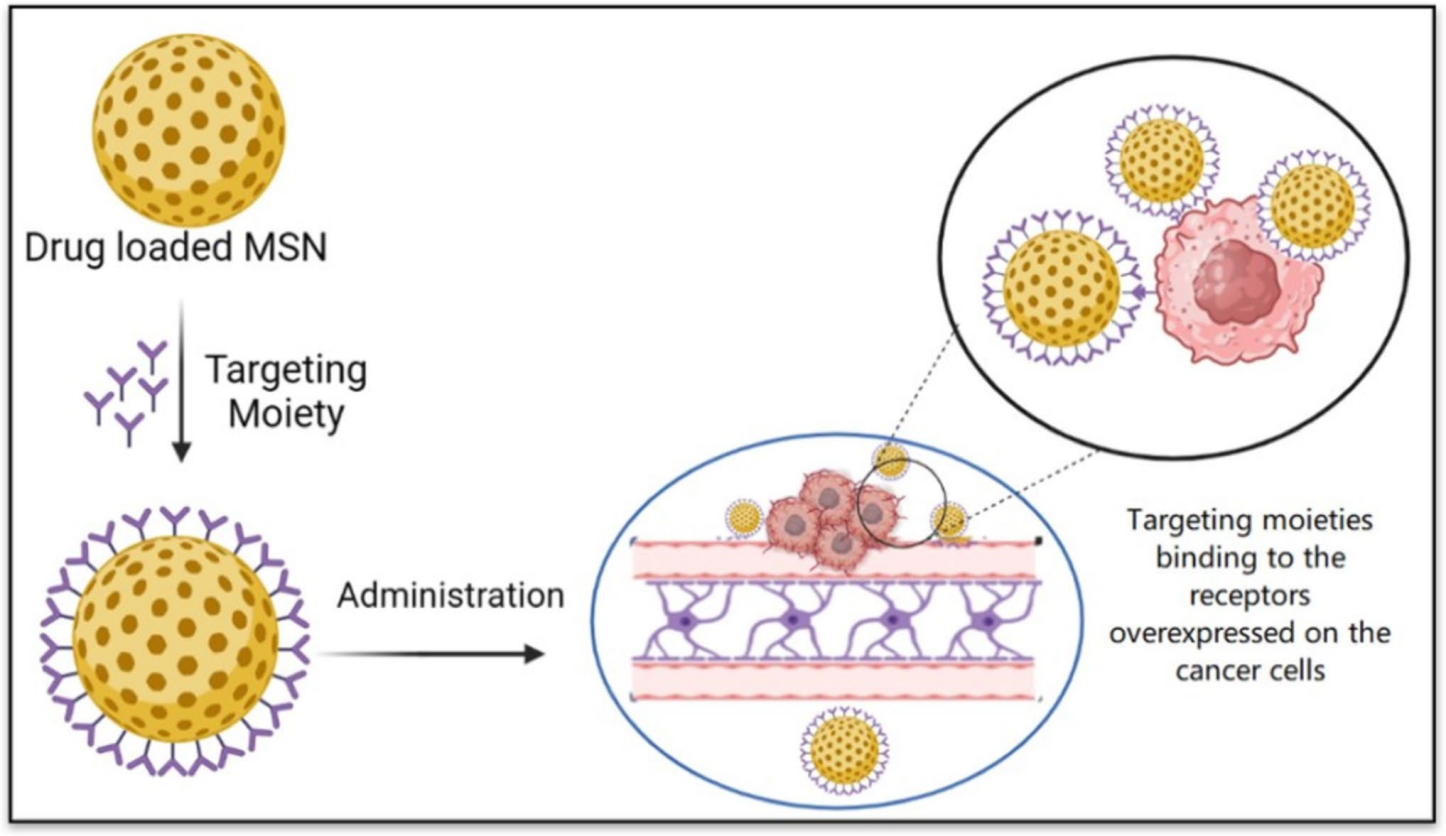
Basic Concept of Surface Modification for Targeting Therapy. This figure shows how MSNs are modified with ligands (e.g., antibodies, peptides) to target GBM cells. Modifications like PEGylation or transferrin conjugation enhance blood–brain barrier penetration and reduce off-target effects

NPs of different compositions, sizes, shapes, surface charges, and surface chemistries can be efficiently transferred across the BBB by optimizing their properties, thus making them specific to the BBB or glioma cells. This modification is done on surface of NPs, allowing them to bind to the target cells more and penetrate barrier, as depicted in Fig. 3 (Mulvihill et al. 2020; Zhao et al. 2020). To increase the specificity of NPs for GBM cells, scientists have investigated the use of ligands that can selectively attach to specific characteristics present on the external membrane of GBM cells(Yeini et al. 2021). Researchers have conducted thorough investigations into ligands such as folic acid (FA), transferrin (Tf), hyaluronic acid (HA), and lactoferrin (Lf) due to their remarkable ability to bind to specific receptors found on the surface of GBM cells. The details of these ligands and their receptor interactions are summarized in Table 1 (Turan et al. 2019).

**Table 1:** Summary of Ligands and Their Receptors in GBM Cells with their Applications

Targeting moiety	Description and mechanism	Target receptor/Feature	Key benefits	Applications
Hyaluronic Acid (HA)	A linear polysaccharide (glycosaminoglycan) that binds to CD44 receptor, which is overexpressed in GBM cells. HA-modified nanoparticles enhance tumor targeting and drug uptake	CD44 receptor	Enhanced uptake by GBM cells, tumor-specific drug release in acidic tumor microenvironment, anti-proliferative effects	HA-FA-coated MSNs delivering 5-FU showed increased cytotoxicity in GBM cells(Curcio et al. 2024)
Folic Acid (FA)	Binds specifically to folate receptors (FR-α, FR-β) overexpressed on GBM cells, enabling receptor-mediated endocytosis of drug-loaded NPs	Folate receptors (FR-α, FR-β)	Targeted delivery to GBM cells, controlled drug release, improved radiosensitization, reduced systemic toxicity	FA-functionalized MSNs delivering cisplatin or valproic acid improved drug efficacy and radiosensitization in GBM models (Zhang et al. 2017)
Transferrin (Tf)	Targets transferrin receptors highly expressed on BBB and GBM cells, facilitating receptor-mediated endocytosis and deeper tumor penetration	Transferrin receptor (TfR)	Enhanced BBB penetration, increased uptake by GBM cells, inhibition of GBM cell migration	Tf-conjugated porous silicon NPs showed enhanced uptake and migration suppression in GBM cells (Sheykhzadeh et al. 2020)
Lactoferrin (Lf)	A glycoprotein similar to transferrin, capable of crossing BBB via receptor-mediated transcytosis; inhibits GBM cell growth by targeting cyclin D1/D4	Lactoferrin receptors on GBM cells	Improved BBB permeability, increased GBM cell uptake, tumor spheroid penetration, induction of apoptosis	Lf-coated ultra-small silica NPs enhanced BBB crossing and apoptosis induction in GBM cells (Janjua et al. 2021)

#### Hyaluronic Acid

HA, referred to as hyaluronan or HA, falls under the category of glycosaminoglycans. It represents a linear polysaccharide composed of a disaccharide structure, specifically alternating (1–4)-β linked D-glucuronic acid and (1–3)-β linked N-acetyl-D-glucosamine residues (Kogan et al. 2007). A composite structure composed of HA deca-saccharide incorporates specific chemical alterations of the glucuronic acid moiety’s carboxylic acid or the N-acetylglucosamine sugar’s C-6 hydroxyl group. Various other alterations of HA have been reported for biomedical applications, including thiol, haloacetate, dihydrazide, aldehyde, tyramine, and huisgen cycloaddition (Burdick and Prestwich 2011). HA is crucial in tumor angiogenesis, tumor growth, cell migration, and progression. CD44, a receptor for HA, is overexpressed in cancer cells. Traditionally, it has been employed as a targeting agent for CD44 to facilitate preferential uptake with enhanced therapeutic efficacy (Hayward et al.).

Curcio et.al. explored MSNs coated with functional biopolymers, hyaluronic acid-Folic acid (HA-FA) and Carboxymethyl chitosan-dopamine, for the development of a targeted delivery system for 5-fluorouracil (5-FU) and silymarine (Si). MSNs synthesized from the sol–gel method were found to have a particle size of around 185 nm, which varied to 227 nm and 238 nm for HA-FA and CMCH-DA coated 5-FU MSNs, respectively, indicating successful coating and dispersion. The drug release profile was evaluated for three pH conditions (5.5, 6.8, and 7.4) to mimic the TME and physiological conditions, which showed that all the formulations had a significantly higher release at pH 5.5, confirming their capability to release drugs in acidic TME, minimizing the premature leakage under normal physiological conditions. Similarly, in vitro cytotoxicity assays performed in MCF-7 (breast cancer cell line) and T98G (GBM cell line) showed a significantly reduced cell viability with HA-FA/5-FU-MSN and CNCH-DA/5-FU-MSN, with HA-FA showing the most pronounced effects. Moreover, it was also found that the HA-FA polymer showed a degree of anti-proliferative activity that could be utilized for a synergistic approach. These results also highlight the efficiency of HA-mediated targeting through CD44 receptor interactions, which is commonly overexpressed in various tumors. The targeted formulations showed an increased internalization and enhanced cytotoxic effects over 24, 48, and 72 h time points. These properties make it particularly suitable for treating aggressive cancers like glioblastoma and breast cancer (Curcio et al. 2024).

CD44 is a transmembrane proteoglycan that can recognize and bind glycosaminoglycan-based biomaterials such as HA and CS. CD44 is activated by binding to its primary ligand, HA, which triggers downstream signaling. Thus, using hyaluronic acid-modified MSNs for targeted drug delivery could be a potential therapeutic strategy for GBM (Yoshida et al. 2012; Du et al. 2022).

#### Folic Acid (FA)

FA demonstrates a remarkable affinity for folate receptors (FR-α and FR-β), transmembrane proteins associated with folic acid. While FR expression in normal tissues remains low, certain tumors demonstrate overexpression of these receptors, rendering them prime targets for anticancer interventions. In the context of GBM, FA serves as a targeting moiety due to its specific binding to folate receptors. This molecular interaction enables folic acid-modified NPs to penetrate tumor cells via receptor-mediated endocytosis, consequently bolstering GBM-targeting capabilities of nano-drug delivery systems (Jaracz et al. 2005).

MSNs functionalized FA represent a promising advancement in targeted drug delivery, particularly for the treatment of glioblastoma, a highly lethal brain tumor. MSNs are highly advantageous due to their unique morphological features, such as large surface areas, adjustable pore sizes, and high biocompatibility, making them ideal carriers for therapeutic agents like cisplatin (Cis-Pt). The Stöber method enables the synthesis of MSNs with uniform sizes and well-defined porous structures, which are critical for controlled drug loading and release. Functionalizing these nanoparticles with FA influences the high affinity of FA for folate receptors, which are overexpressed in many cancer cells, including GBM. This selective targeting ensures that Cis-Pt is released specifically at the tumor site, enhancing the cytotoxic effect on GBM cells while minimizing systemic side effects. Detailed characterization techniques, including SEM, AFM, and TEM, confirm the successful synthesis and functionalization of MSN-FA/Cis-Pt, showing uniform spherical nanoparticles with significant surface areas and porosity. The in vitro release studies indicate a sustained and controlled release of Cis-Pt, with mathematical modeling suggesting that the Higuchi model best describes the release kinetics. This targeted delivery system not only improves drug efficacy but also offers a strategic approach to overcome the limitations of conventional GBM therapies, highlighting its potential for further development and clinical application (Ortiz-Islas et al. 2021).

Zhang et. al. developed a novel strategy for improving radiotherapy efficacy utilizing FA-targeted MSN loaded with valproic acid for the treatment of GBM. This is done to achieve tumor-specific delivery and pH-responsive release, enhancing the radiosensitization while minimizing the systemic toxicity. The developed nanoparticles were approximately 180 nm in size, with release kinetics depicting almost 76% drug release within 15 h at acidic pH, representing the TME, while the release at physiological pH was minimal, suggesting a pH-responsive behavior due to the prevention of premature drug leakage. Cell viability study showed that the developed nano-systems had a significantly better cytotoxicity profile compared to free VPA and blank MSNs. The IC50 values for developed nanoparticles in C6 cells and U87 cells at 12 h were 58,456 mg/L and 52,923 mg/L, respectively, significantly better than free VPA. Flow cytometry showed negligible apoptosis in the absence of radiation, confirming minimal cytotoxicity of the VPA-MSNs alone. Upon 4 Gy irradiation, apoptosis rates increased significantly to 9.75% in C6 and 8.71% in U87 cells treated with VPA-MSNs, compared to 0.68% and 1.01% in the VPA-only groups. Moreover, at 8 Gy, apoptosis was further elevated, reaching 13.76% in C6 and 11.54% in U87 under acidic pH (6.0), demonstrating enhanced efficacy via pH-triggered release. Clonogenic assay revealed that the SER for VPA-MSNs at pH 6.0 was 1.714, significantly higher than VPA alone (1.091), MSNs (1.197), and VPA-MSNs at neutral pH (1.507), indicating superior radiosensitization. Western blotting showed increased levels of pro-apoptotic markers and reduced Bcl-2 in VPA-MSN + radiation groups, especially under acidic conditions, confirming activation of apoptosis pathways (Zhang et al. 2017). Researchers have already developed folic acid-conjugated biodegradable NPs coated with poly (ethylene glycol) (PEG) for targeted therapy to tumor cells. The NPs have shown precise binding to the folate-binding protein, suggesting their potential as a selective drug carrier for tumor cells (Kim et al. 2005). FA-conjugated MSNs (FA@MSN) exhibit significant promise as a therapeutic strategy for GBM. Through MSN surfaces functionalization with FA molecules, FA@MSN NPs achieve specific targeting of GBM cells characterized by folate receptor overexpression. This targeted delivery system enables the selective accumulation of FA@MSN within GBM tumors, while minimizing uptake by normal cells, thereby reducing toxicity concerns. The incorporation of folic acid onto MSN facilitates precise binding to folate receptors on cancer cell surfaces, augmenting the FA@MSN internalization into GBM cells and consequently elevating the concentration of chemotherapeutic agents within tumor cells. In comparison to non-functionalized NPs or free drugs, FA@MSN demonstrates the potential to deliver higher doses of chemotherapeutic agents directly to tumor cells, thereby potentially improving treatment efficacy.

The mesoporous structure of FA@MSN enables effective loading of the drug, along with controlled release of the enclosed chemotherapeutic agents, which can be finely tuned to achieve a consistent and controlled drug release pattern. As a result, it enables prolonged exposure of tumor cells to therapeutic agents, enhancing their effectiveness. The larger surface area of FA@MSN provides opportunities for additional modifications. For example, imaging agents are attached to the surface of MSNs, enabling real-time monitoring of the response of the tumor to therapy. Therapeutic payloads, such as gene therapy vectors or immunomodulatory, can also be incorporated into the MSNs’ structure, functionalized modifications, allowing combination therapies or targeted immune modulation. As a result, FA@MSN has the prospective to enhance treatment outcomes and overcome the hurdles related to GBM therapy by selectively delivering chemotherapeutic agents to tumor cells while minimalizing off-target effects.

#### Transferrin (Tf)

Passive targeting strategies have restraints in regards to specificity, and the outcome of NPs is primarily determined by their physicochemical properties. To address these limitations, targeted approaches utilizing receptor-mediated endocytosis are extensively employed. Transferrin receptor (TfR) targeting is effective in GBM due to its overexpression in tumor cells and the BBB. NPs functionalized with Tf, antibodies, or targeting peptides penetrate deeper into tumor models, demonstrating enhanced antiproliferative activity. In mice with intracranial tumors, Tf-functionalized NPs accumulate effectively in brain tumor tissue, improving drug efficacy and increasing survival rates.

Researchers have effectively developed porous silicon NPs (pSiNP) conjugated with Tf and examined their effectiveness in inhibiting the GBM cells migration. Employing a microfluidic-based migration chip designed to replicate brain tissue, they observed that Tf@pSiNP exhibited remarkable stability, biocompatibility, and demonstrated an enhanced uptake by GBM cells via receptor-mediated internalization. Exposure of GBM cells to Tf@pSiNP led to significant migration suppression within restricted microchannels, potentially achieved by destabilizing focal adhesions and reducing cellular volume. These findings illuminate the encouraging possibilities of Tf@pSiNP as a disruptive strategy in GBM therapy, synergistically combining the abilities of migration inhibition and targeted drug delivery. Transferrin was successfully conjugated onto porous silicon NPs (pSiNP), resulting in Tf@pSiNP having an average size of 182 ± 0.8 nm, which is determined by DLS. Cryo-TEM imaging confirmed the plate-shaped structure of Tf@pSiNP. As a comparative measure, pSiNP modified with bovine serum albumin (BSA@pSiNP) displayed analogous attributes, including size (172 ± 4 nm) and zeta potential (-9.9 ± 4.4 mV). Confocal microscopy analysis revealed that Tf@pSiNP demonstrated enhanced uptake into U87 cells, a type of GBM cells, compared to BSA@pSiNP. Cryo-TEM imaging further confirmed the internalization of Tf@pSiNP in U87 cells. Biocompatibility studies using an adenosine triphosphate (ATP) bioluminescence assay demonstrated that Tf@pSiNP and BSA@pSiNP did not adversely affect cell viability or ATP levels in U87 cells. Interestingly, cells exposed to Tf exhibited higher ATP levels, possibly attributed to increased metabolic activity facilitated by Fe^2+^ uptake. Importantly, Tf@pSiNP exhibited enhanced uptake by GBM cells without causing toxicity or reducing ATP levels. Utilizing a microfluidic chip with narrow topographical constraints, it was opined that therapy with Tf@pSiNP resulted in a reduction in GBM cell migration through microchannels. These findings suggest that the observed effects may be attributed to a decrease in focal adhesion maturation and resistance to cell volume reduction, both of which play crucial roles in GBM cell migration. This data indicated the potential use of Tf@pSiNP as therapeutic strategy to restrict GBM cell migration (Sheykhzadeh et al. 2020).

#### Lactoferrin (Lf)

A type of glycoprotein called Lf, belonging to the family of Tf, can bind and transport iron. Researchers have discovered that GBM cells possess receptors for Lf, suggesting their potential to facilitate the passage across the BBB via transcytosis and enter GBM through receptor-mediated signaling pathways. Additionally, studies have depicted that Lf can decrease the growth of malignant GBM cells by inhibiting cyclin D1 and D4 (Zhang et al. 2018a).

Cyclin D1 is pivotal in cell cycle progression and is often upregulated in various tumor types, driving cancer advancement. Targeting the degradation of cyclin D1 presents a promising approach for cancer therapy (Chen and Li 2022). The researchers successfully synthesized and modified ultra-small silica NPs (USLP) by incorporating doxorubicin. They also coated the USLP with Lf, resulting in Lf-coated USLP (Lf@USLP). The Lf@USLP NPs exhibited enhanced permeability through the BBB, increased uptake by GBM cells, improved penetration into tumor spheroids, and enhanced ability to induce apoptosis. The key objective of the study was to synthesize ultra-small silica NPs with specific characteristics: approximately 30.74 nm in size, large pores, and a negative surface charge to facilitate effective delivery of medications to brain tumors. The study effectively showcased the development of these NPs and their potential for precise drug delivery to brain tumors, using in vitro BBB model. Lf@USLP displayed the potential to traverse the brain endothelial cell monolayer and internalize into brain tumor cells. The small size of uncoated USLP hindered its detection in GBM cells, likely attributed to their restricted ability to penetrate the BBB or undergo cellular uptake. Notably, further research indicated that USLP exhibited satisfactory uptake in GBM cells, while the influence of lactoferrin on nanoparticle uptake remained inconclusive. Moreover, utilizing a 3D tumor spheroid model, both coated and uncoated USLP demonstrated the ability to penetrate the tumor spheroid, with the lactoferrin coating facilitating enhanced BBB penetration compared to uncoated USLP. Importantly, Lf@USLP successfully traversed the brain endothelial cell monolayer and internalized into brain tumor cells. To mitigate premature release into the bloodstream and minimize systemic exposure to critical tissues, sustained release systems such as liposomal-coated doxorubicin (Doxil®) have been employed, which can effectively reduce cardiotoxicity(Safra et al. 2000). Further in the research studies, the USLP were loaded with DOX which portrayed controlled release, with slower release at thr pH 7.4 (simulating plasma) alongside faster release at pH 5.5 (mimicking GBM microenvironment and lysosome conditions) (Janjua et al. 2021). Lf coating further slowed the release. USLP-DOX induced apoptosis in GBM cells, with higher levels observed for Lf@USLP-DOX. In 3D tumor spheroid models, USLP-DOX reduced tumor size, particularly with negatively charged USLP-PO3 formulations. Lf coating improved nanoparticle penetration and decreased tumor growth. As a result, by using surface-modified USLP-based delivery systems, the effectiveness of DOX can be enhanced against GBM by overcoming limitations such as BBB passage and tumor cell targeting(Janjua et al. 2021).

Additionally, the RGD peptide, a short amino acid sequence, has been studied as a ligand for targeting GBM cells. The RGD peptide has an affinity towards the αVβ3 integrin protein, which is predominantly present on GBM cells’ surface. It is often used with polymeric NPs, which are composed of long chains of repeating subunits and can be engineered to have specific properties for drug delivery(Temming et al. 2005; Pinheiro et al. 2021). Other strategies that might enhance the TMZ effect include antiangiogenic therapy, tumor-treating fields, and immune checkpoint inhibitors. As previously stated, angiogenesis is the evident defining trait of many tumors, comprising adult brain tumors (GBM), that distinguishes GBM from healthy brain tissues. Thus, the most effective method for treating patients with GBM is now deemed to be anti-angiogenesis therapy. Given that VEGF has been established to have a pivotal role in the angiogenesis of GBMs, the most effective approach to prevent the occurrence of GBMs in patients is to suppress the expression of VEGF (Das and Marsden 2013; Wang et al. 2017; Ahir et al. 2020; Zhu et al. 2021).

Researchers have recently identified a potential new treatment for GBM called vasculogenic mimicry (VM), which involves tumor cells forming blood vessel-like structures to obtain oxygen and nutrients independently of the host blood supply(Fernández-Cortés et al. 2019). These structures are not formed by endothelial cells and have different functional properties than normal blood vessels, and they are formed by tumor cells that have taken on a stem cell-like phenotype and can differentiate into various cell types. Targeting VM may be a promising strategy for treating GBM, as it could limit the blood supply to the tumor and make it more susceptible to traditional treatments (Folberg et al.; Frickenstein et al. 2021).

#### Other ligands

In addition to hyaluronic acid, folic acid, transferrin, and lactoferrin, several other ligands have been explored for their ability to enhance the specificity of MSNs in targeting GBM cells. These ligands exploit overexpressed receptors or proteins on GBM cells or the BBB, facilitating precise drug delivery and imaging. This section reviews key ligands, including RGD peptide, chlorotoxin, Angiopep-2, interleukin-13 (IL-13), epidermal growth factor receptor (EGFR/EGFRvIII), and aptamer AS1411, highlighting their mechanisms and potential in MSN-based GBM therapy.

*RGD Peptide:* The RGD peptide, a short amino acid sequence (Arg-Gly-Asp), targets αVβ3 integrin, which is overexpressed on GBM cells and tumor-associated vasculature. This ligand promotes receptor-mediated endocytosis, enhancing nanoparticle uptake by GBM cells (Xu et al. 2019). Xu and his colleagues developed a photo-triggered one-pot cycloaddition method for the conjugation of RGD-Acrk peptides to biodegradable mesoporous silica nanoparticles (bMSN), forming the fluorescent nanoprobe bMSN@T2-RGD-Acrk (Xu et al. 2019). This system employs a tetrazole–alkene cycloaddition to create a pyrazoline fluorophore emitting at 550 nm, aimed at biocompatible, non-toxic, site-specific labeling for in vivo tumor imaging with high signal-to-background ratios (STBRs). The precursor T2 was synthesized in four steps with 40% yield, exhibiting UV absorption at 287 nm that shifted to 369 nm upon photoactivation, confirming fluorescent ring formation. The cycloaddition was optimized under 254 nm UV light for 0.5–2 h, yielding 54% T2-AM after 2 h. The key conjugation step involved reacting 10.8 µmol of T2 with 1.5 mg of bMSN, followed by attachment of the RGD-Acrk peptide to form bMSN@T2-RGD-Acrk nanoparticles. This conjugation enabled targeted delivery via αvβ3/αvβ5 integrins, resulting in exceptional stability (> 5 days without aggregation or size changes) in PBS and 10% FBS at 25–37 °C. In vitro studies with 4T1 murine breast cancer cells (2 × 10^4^ cells/dish) demonstrated strong cytoplasmic and membrane green fluorescence after 4-h dark incubation, with a 24-h pre-incubation using free RGD-Acrk peptide significantly reducing intensity, confirming specific receptor-mediated uptake and targeting efficacy. MTT assays on bMSN@T2-RGD-Acrk and its components (bMSN, T2, RGD-Acrk) showed > 90% cell viability, underscoring non-toxicity. In vivo, female BALB/c mice with 4T1-luciferase tumors exhibited rapid nanoprobe localization within 30 min, peaking at 4 h, with the targeted group achieving a markedly higher STBR (2.71 ± 0.33) versus the non-targeted group (1.11 ± 0.26), highlighting enhanced tumor selectivity due to conjugation. Histology verified accumulation primarily in tumor tissues. Biocompatibility tests (50 mg/kg injection) yielded 95% survival after 14 days, with no inflammation or damage. Overall, the conjugation process produced stable, targeted fluorescent nanoprobes with superior specificity, offering a promising platform for early cancer diagnosis and real-time imaging.

*Chlorotoxin:* Chlorotoxin, a 36-amino-acid peptide derived from scorpion venom, selectively binds to matrix metalloproteinase-2 (MMP-2) and integrins overexpressed on GBM cells. Its ability to target GBM cells with high specificity makes it an attractive ligand for nanoparticle functionalization (Wang et al. 2020). Chlorotoxin-modified MSNs have been used to deliver therapeutic agents and imaging probes, enhancing tumor-specific uptake and improving diagnostic accuracy. Mundžić and group developed chlorotoxin (CTX)-conjugated PLGA–PEG nanoparticles for targeted glioma therapy (Mundžić et al. 2025). CTX, a peptide from scorpion venom, was covalently linked to the nanoparticle surface to target matrix metalloproteinase-2 and chloride channels overexpressed in glioma cells. The nanoparticles had a mean diameter of 83 ± 7 nm, a polydispersity index of 0.12 ± 0.03, and a zeta potential of − 21.4 ± 1.6 mV, with 79.5 ± 4.2% encapsulation efficiency and 8.6 ± 1.3% drug loading. In vitro, they released ~ 75% of the drug within 72 h. CTX conjugation enhanced cellular uptake in U87MG and C6 glioma cells by 3.5-fold after 4 h and reduced cell viability to 42% ± 3.8% compared to 61% ± 4.5% for non-targeted nanoparticles, with a twofold lower IC₅₀. In vivo, in U87MG glioma-bearing mice, CTX nanoparticles (10 mg/kg) showed a tumor-to-normal brain fluorescence ratio of 4.2 ± 0.7 versus 1.3 ± 0.2 for non-targeted nanoparticles, reduced tumor volume by 63% over 21 days, and extended median survival by 1.8-fold. Biodistribution confirmed tumor-specific accumulation with no toxicity in major organs. The CTX-functionalized nanoparticles offer a biocompatible, targeted nanotherapeutic for glioma treatment.

*Angiopep-2:* Angiopep-2 is a peptide ligand that targets low-density lipoprotein receptor-related protein 1 (LRP1), which is overexpressed on both GBM cells and brain endothelial cells forming the BBB (Zhu et al. 2021). This dual-targeting capability makes Angiopep-2 particularly effective for facilitating nanoparticle transcytosis across the BBB and subsequent uptake by GBM cells (Xin et al. 2011). Tao and colleagues developed angiopep-2-conjugated liposome–silica hybrid nanovehicles (ANG-LP-PAA-MSN@ATO) for targeted, pH-triggered delivery of arsenic trioxide (ATO) to glioma, addressing poor BBB permeability and systemic toxicity (Tao et al. 2019). Angiopep-2 conjugation enhanced performance. Cellular uptake of ANG-LP-PAA-MSN@FITC was 2.16-fold higher in C6 glioma cells and 2.56-fold higher in brain endothelial cells compared to non-targeted LP-PAA-MSN@FITC. Cytotoxicity was greater, with an IC₅₀ of 5.56 ± 0.24 mM for ANG-LP-PAA-MSN@ATO versus 6.41 ± 0.11 mM for ATO-Sol. BBB penetration was 7.70 ± 0.48%, 2.53-fold higher than PAA-MSN@ATO and 2.02-fold higher than LP-PAA-MSN@ATO. In vivo, ANG-LP-PAA-MSN@ATO extended ATO’s half-life in glioma tissue by 2.34-fold and increased AUC by 9.5-fold compared to free ATO. Targeting efficiency (Te) was 0.250 versus 0.069–0.149 for non-conjugated carriers. Tumor uptake reached 4.6 ± 2.6% ID/g versus 0.8 ± 0.3% ID/g for free ATO, reducing tumor volume to 27.43 ± 11.92 mm^3^ and extending median survival to 49.5 days from 28 days with ATO-Sol. Angiopep-2 conjugation enabled receptor-mediated BBB transport, enhanced uptake, prolonged circulation, and improved glioma targeting, making ANG-LP-PAA-MSN@ATO a promising nanotherapeutic for glioma.

The epidermal growth factor receptor (EGFR) and its mutant variant EGFRvIII are frequently overexpressed in GBM, positioning them as key targets for ligand-based therapies. Nanoparticles functionalized with antibodies or peptides specific to EGFR/EGFRvIII enhance tumor targeting, cellular uptake, and the delivery of chemotherapeutics or imaging agents (Montano et al. 2011). Other promising ligands include Aptamer AS1411, a stable DNA aptamer that binds nucleolin, a protein overexpressed on GBM cell surfaces, enabling high-specificity nanoparticle functionalization (Bie et al. 2022); and interleukin-13 (IL-13), a cytokine that selectively targets interleukin-13 receptor alpha-2 (IL-13Rα2), a tumor-restricted antigen abundant in GBM but minimal in normal brain tissue (Gao et al. 2014). Additional ligands such as RGD peptide, chlorotoxin, and Angiopep-2 further expand targeting options by exploiting receptors or proteins overexpressed in GBM or BBB transporters, thereby improving nanoparticle penetration, uptake, and therapeutic efficacy. Collectively, these ligands, EGFR/EGFRvIII, AS1411, IL-13, RGD, chlorotoxin, and Angiopep-2, strengthen the potential of MSNs for multimodal GBM strategies, including targeted drug delivery alongside imaging, photodynamic therapy, or radiosensitization. However, limited data exist on integrating these ligands with MSNs for GBM or brain tumor treatment, underscoring a critical research gap. Further studies are needed to optimize ligand-MSN conjugates, evaluate long-term safety, and assess clinical viability in overcoming GBM’s formidable challenges.

## Various silica nanoparticles used

The BBB has a natural filter that limits the number of compounds that can enter the CNS. This poses a challenge in drug development and requires the synthesis of novel drug delivery system (NDDS). One promising solution is the use of silica NPs, which can be customized to fit different applications and manufactured in various sizes thanks to their versatile surface properties (Jeelani et al. 2020). These NPs have the potential to revolutionize drug delivery system (DDS) to the brain. In this regard, silica NPs may be classified based on their surface morphology, shape, and pore morphology (Mehmood et al. 2017). Among these are MSN, non-porous silica (NPSN), hollow mesoporous silica (HMSN), and core–shell silica. Each type of silica nanoparticle can be tailored to suit specific purposes. MSN possesses an array of adaptable and desirable properties, including high drug loading ability, pore size variability, bulk, and biocompatibility (Möller and Bein 2017; Farjadian et al. 2019; Khan et al. 2019). It has been utilized as an excipient in table pharmaceutical synthesis for over 50 years and has been deemed "generally recognized as safe" (GRAS) by the FDA (Farjadian et al. 2019). MSN, also known as a nano sponge, is capable of adsorbing large amounts of molecules dissolved in solution because of its large surface area. Approximately the same surface area as an American football field is covered by 5 g of MSNs (Vallet-Regí et al. 2018; Lai et al. 2023). It is possible to arrange the pores in a variety of orientations, such as MCM-41, MCM-48, radial, cubic, wormlike, etc. In addition to simplifying the encapsulation process, this modification of the pore arrangement is intended to regulate the rate at which cargo is released(Kazemzadeh et al. 2022).

## Method of preparation of silica nanoparticles

**Fig. 4:**
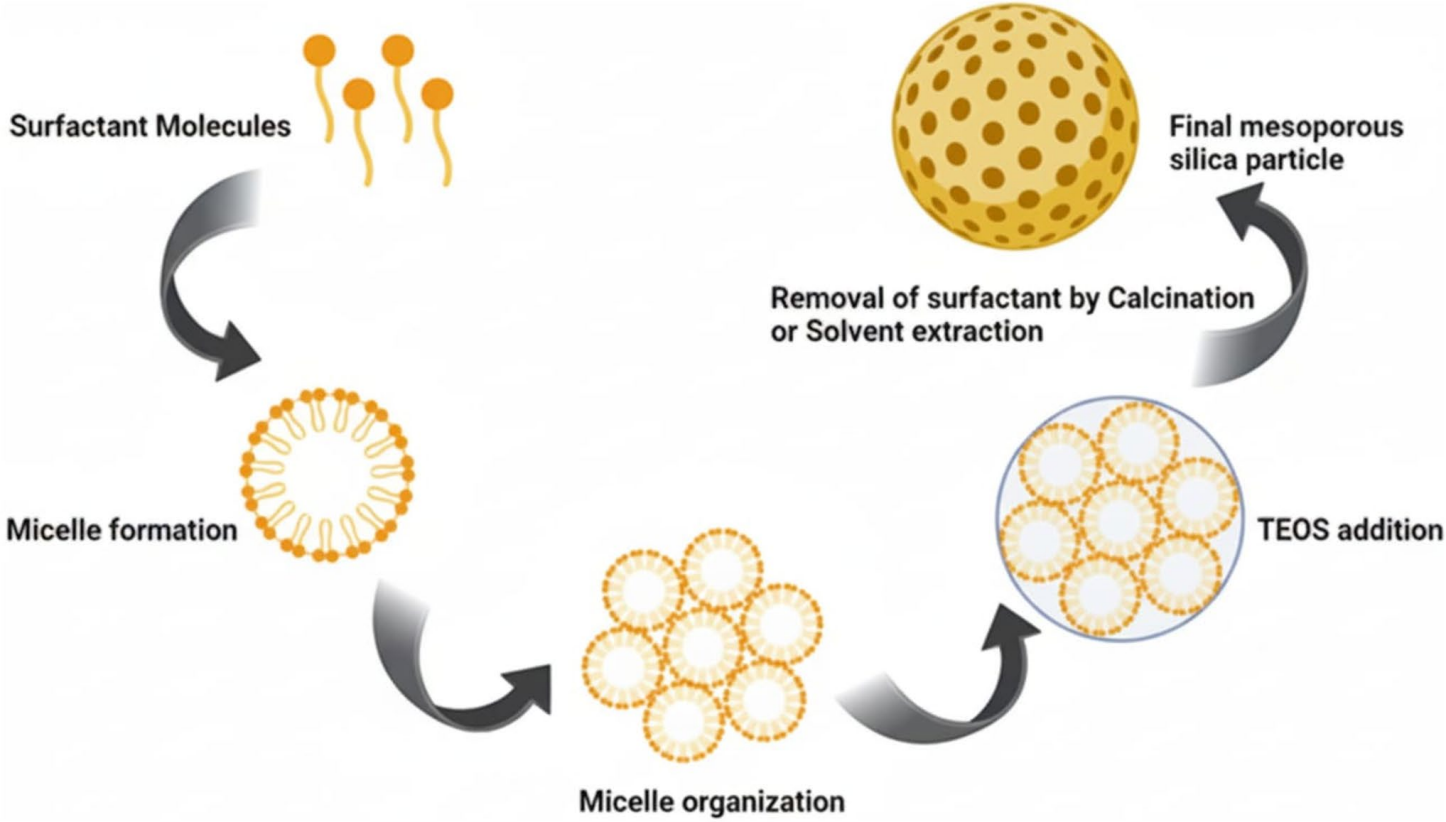
Synthesis of Mesoporous Silica Nanoparticles (MSNs). This figure illustrates multiple methods for synthesizing MSNs for GBM therapy. The sol–gel method involves hydrolysis and condensation of silica precursors (e.g., tetraethyl orthosilicate) with surfactant templates (e.g., CTAB) to form ordered mesoporous structures, followed by template removal. Alternative methods include hydrothermal synthesis, adjusting temperature and pressure for larger pores, and emulsion-based synthesis for uniform particle size. The figure highlights surface functionalization (e.g., with amines or PEG) to optimize MSNs for drug loading and targeted delivery, emphasizing control over pore size and particle morphology

There have been numerous developments of MSNs over the past few years. The synthesis of MSNs can be tweaked by using different polymers/surfactants and silica sources and concentrations. Most synthetic procedures for producing mesoporous silica materials rely on surfactants to direct their structure. Various techniques are employed in the synthesis of these materials, including fast self-assembly, hydrothermal synthesis, sol–gel synthesis, modified aerogel methods, template synthesis, and soft and hard templating method (Narayan et al. 2018; Ghaferi et al. 2021). Amidst the various methodologies employed, the sol–gel technique that employs tetraethyl orthosilicate (TEOS) and surfactant template, stands out as a frequently employed procedure to manufacture MSNs. The surfactant micelles serve as a model to form channels within the silica structure, resulting in MSNs after the gelation process. The surfactant is later eliminated through calcination or solvent removal, leaving behind the desired particles (Dixit et al. 2016). Stöber method, the reverse microemulsion method, and the template-free method. The Stöber method involves hydrolyzing and condensing TEOS in the presence of a stabilizing agent and a basic solution, as portrayed in Fig. 4. The resulting uniform silica NPs can be functionalized to create MSNs (Ren et al. 2020).

The reverse microemulsion method, on the other hand, employs a water-in-oil microemulsion system where TEOS is hydrolyzed and condensed in water droplets. The surfactant molecules around the droplets determine the shape as well as the size of the particles, which are then purified by washing to remove any impurities(Rosenberg et al. 2019). Finally, in the template-free method, a basic solution and a cosolvent are used to facilitate hydrolysis and condensation of TEOS. The morphology of MSNs is regulated by adjusting the cosolvent, and the resulting particles are calcined to produce MSNs (Imoisili and Jen 2022).

The Aerosol-assisted self-assembly (AASA) method creates MSNs using an aerosol generator to produce a fine mist of a silica precursor solution containing surfactants and other additives. Subsequently, the mist undergoes a drying and calcination process, resulting in the formation of NPs characterized by equal pore size distribution and substantial surface area. The method of synthesis using microwave assistance involves utilizing a microwave reactor to quickly heat the reaction mixture, facilitating hydrolysis and condensation of silica predecessors. This process results to the formation of MSNs with a well-defined pore structure and a substantial surface area (98–100). An oil-in-water emulsion system containing a silica precursor and a surfactant is employed in the emulsion-templated synthesis method. The emulsion droplets act as templates for MSNs formation. After reaction, surfactant is removed, and resulting NPs are washed and calcined to create MSNs (Gustafsson and Holmberg 2017; Meaney et al. 2017).

## Applications

**Fig. 5:**
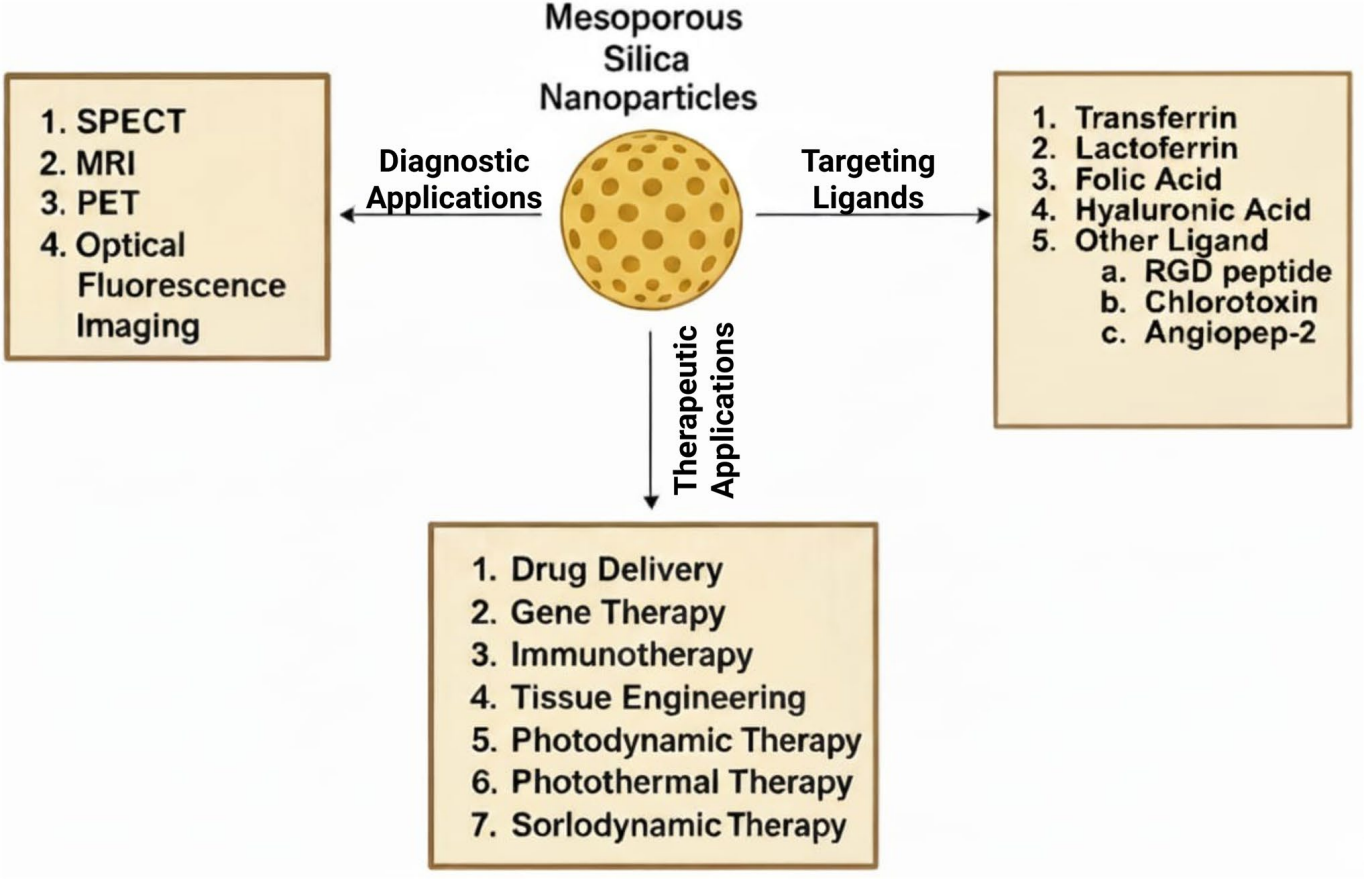
Diagrammatic Representation of Applications of MSNs. This figure illustrates MSN applications in GBM therapy, including drug delivery, imaging (e.g., MRI contrast), and combination therapies like photodynamic and sonodynamic treatments. It emphasizes MSNs’ versatility in addressing GBM challenges

The remarkable structural properties of MSNs have sparked great interest within the scientific community. As a result, MSNs have become highly sought after for numerous applications, like drug delivery, adsorption, catalysis, as well as sensing. The tunable pore size and large surface area of MSNs make them efficient drug carriers and catalysts, while their ability to be easily functionalized allows for tailored applications in various fields. The numerous patents filed on MSNs demonstrate the significant interest and potential for their use in biomedical applications, biosensors, imaging, and their utility as adsorbents, as depicted in Fig. 5.

### Therapeutic applications

**Fig. 6:**
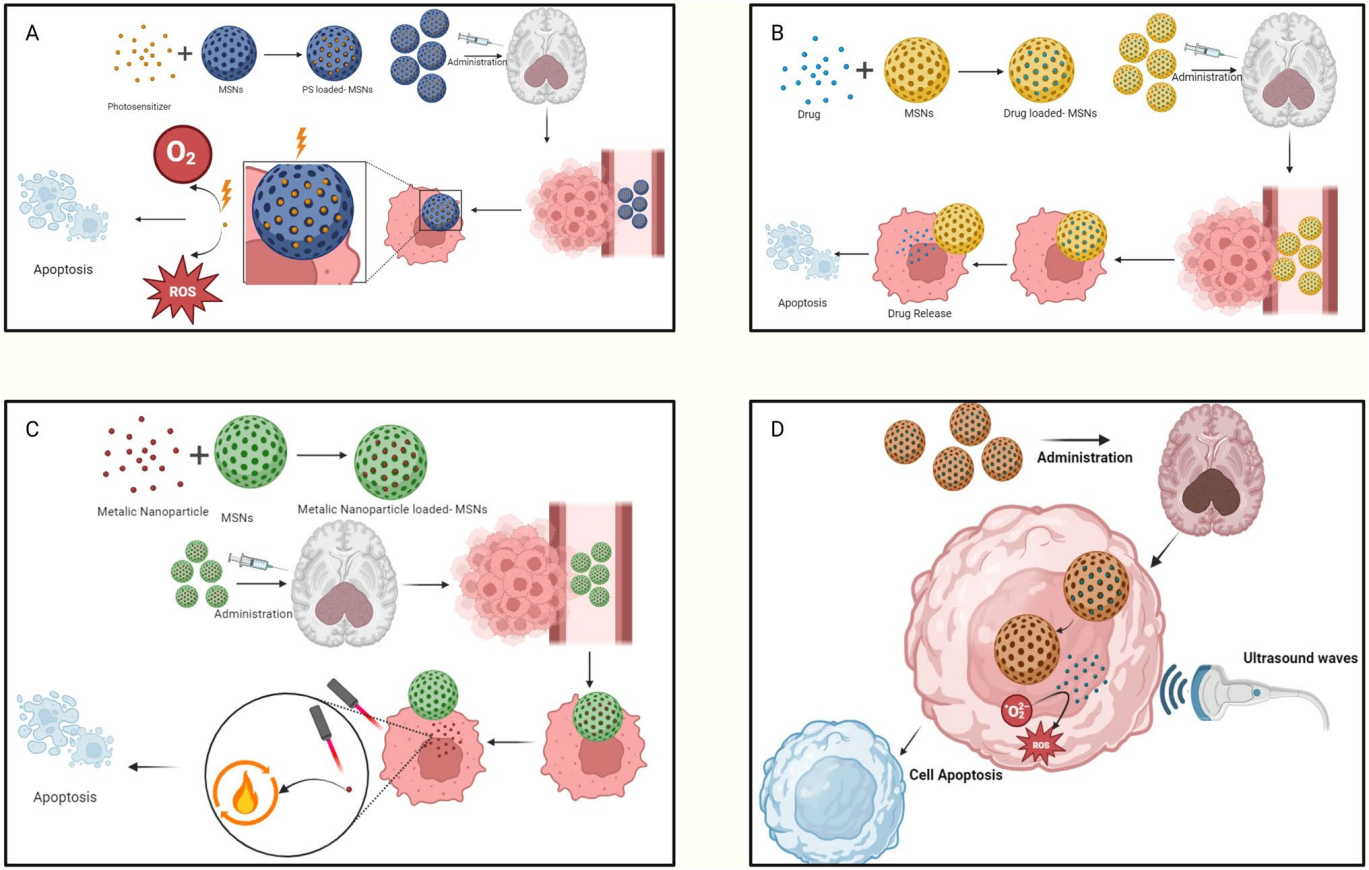
A Mechanism of Action of Drug-Loaded MSNs: This figure shows drug-loaded MSNs crossing the BBB to deliver chemotherapeutics (e.g., temozolomide) to GBM cells. It depicts controlled drug release via intracellular stimuli (e.g., pH), enhancing efficacy and reducing toxicity. **B** Mechanism of Action of Photodynamic MSNs: This figure illustrates photodynamic therapy using MSNs loaded with photosensitizers. Light activation generates reactive oxygen species, inducing GBM cell apoptosis. It highlights targeted delivery and light-based precision therapy. **C** Mechanism of Action of MSNs for Photothermal Therapy: This figure depicts MSNs loaded with metallic nanoparticles (e.g., gold) for photothermal therapy in GBM. It shows targeted delivery to GBM cells, where near-infrared light absorption by metallic nanoparticles generates heat, inducing tumor cell death. The figure highlights MSNs’ role in enhancing tumor-specific heating and minimizing damage to surrounding tissues. **D** Mechanism of Action of MSNs for Sonodynamic Therapy: This figure explains sonodynamic therapy using MSNs loaded with sonosensitizers. Ultrasound triggers reactive oxygen species production, causing GBM cell death. It highlights MSNs’ role in targeted delivery and deep tissue penetration

The distinctive attributes of MSNs, including their tunable particle size and versatile surface chemistry, render them exceptionally promising for advanced therapeutic applications, particularly in the treatment of GBM. Multimodal therapeutic approaches, integrating techniques such as chemotherapy, photodynamic therapy, gene delivery, radiotherapy, and immunotherapy, are essential for GBM management, enabling synergistic effects, targeted drug release, and enhanced penetration across the blood–brain barrier, with MSNs facilitating precise co-delivery of therapeutic agents, stimuli-responsive activation, and real-time monitoring to optimize treatment efficacy and overcome tumor heterogeneity. Figure 6 shows the mechanism of action of a few of the therapies.

#### Chemotherapy

The substantial interest in MSNs stems from their potential to serve as efficient drug delivery vehicles for patients with a broad range of diseases. In another study by Tsung-I Hsu and colleagues developed docetaxel (DTX) loaded MSN for the treatment of TMZ-resistant GBM. The study explores the use of PEGylated MSN for targeted delivery of DTX to overcome TMZ resistance and non-specificity in GBM therapy. This system demonstrated excellent biocompatibility and no hemolytic activity in vitro studies. The cellular uptake and anti-proliferative effects of nanoparticles induced significant apoptosis in TMZ-resistant U87MG cells, with a lower IC50 value (17.8 ng/mL) compared to free DTX, which was about 33 ng/mL. Moreover, the developed nanoparticles increased early apoptosis to around 18% and late apoptosis to 4.5%, which indicated improved delivery and therapeutic efficacy. Similarly, in vivo imaging depicted that these nanoparticles preferentially accumulated in tumor region, indicating that this nanosystem effectively permeate the BBTB. The survival was prolonged by 50% in orthotopic glioma mouse models upon the administration of the developed nanoparticles. It was also found that this reduced tumor volume and side effects in comparison to free drug. The activation of apoptotic pathways was the mechanism involved which was evidenced by elevated levels of cleaved caspase-3 and PARP, suggesting improved therapeutic efficacy and reduced off-target effects (Hsu et al. 2024).

**Fig. 7:**
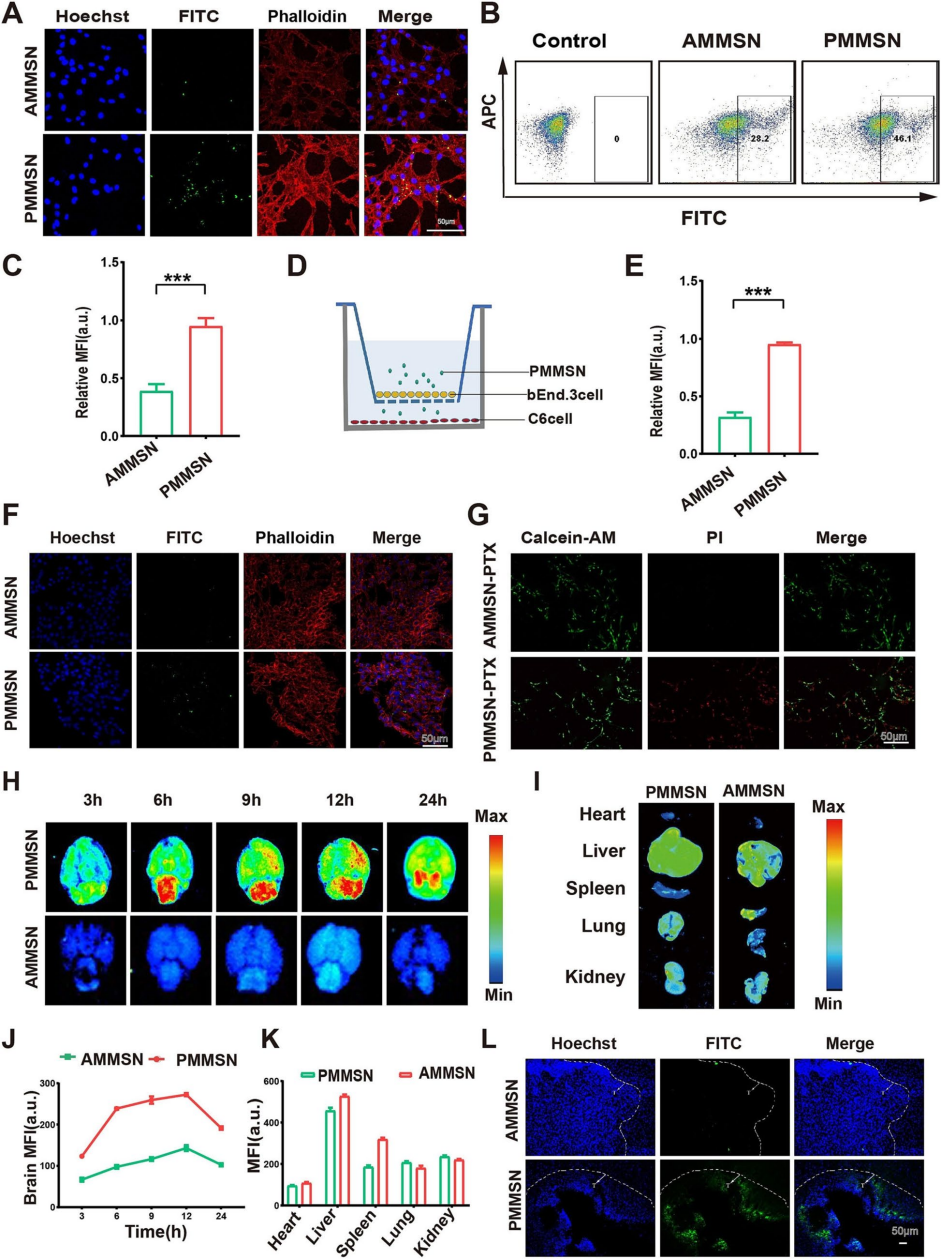
Cellular Uptake and Targeting Study. **A** Fluorescence images of PMMSN and AMMSN uptake by C6 cells. Scale: 50 μm. **B** Flow cytometry analysis of PMMSN and AMMSN uptake by C6 cells. **C** Quantification corresponding to panel A. **D** Transwell diagram for the in vitro blood–brain barrier (BBB) model. **E** Quantification related to panel F. **F** Fluorescence images of PMMSN and AMMSN uptake in the Transwell model. Scale: 50 μm. **G** Fluorescent images of live (green, Calcein-AM) and dead (red, PI) C6 cells treated with PMMSN-PTX and AMMSN-PTX. Scale: 50 μm. **H** Brain fluorescence imaging of glioma-bearing mice after FITC-labeled PMMSN and AMMSN injection via the caudal vein. **I** Fluorescence imaging of the heart, liver, spleen, lung, and kidney 12 h post-injection. **J** Quantification corresponding to panel H. **K** Quantification related to panel I. **L** Fluorescence distribution of FITC-labeled PMMSN and AMMSN in glioma tumor tissue after 12 h. Scale: 50 μm. Adapted with permission from (Liu et al. 2024)

Ying Lui and colleagues developed a nanodrug delivery system using plasma complex component functionalized manganese-doped MSN (PMMSN) loaded with paclitaxel (PTX) for targeted glioma therapy. Figure 7 shows the assessment of targeting of the plasma component conjugated nanoparticles, which showed significantly higher cellular uptake and displayed better targeting efficacy than alginate conjugated nanoparticles, with a higher fluorescence intensity in brain tumor cells and tissues after 3 -24 h, while reaching maximum at 12 h. Similarly, flow cytometry and imaging studies confirmed the enhancement of BBB permeability by PMMSN, resulting in a stronger proapoptotic effect on glioma cells and enhanced tumor targeting and drug accumulation. Moreover, the Oxidative stress evaluation showed that PMMSN substantially enhanced oxidative stress in tumor cells, as suggested by a marked rise in ROS production observed using fluorescence microscopy and quantified by higher MDA levels and lower SOD and GSH-Px levels compared to control and PMSN. Additionally, in animal studies, DHE staining exhibited notably higher fluorescence intensity in tumor tissues, confirming the increased ROS concentration. Furthermore, apoptosis study in C6 glioma cells PMMSN-PTX demonstrated a potent pro-apoptotic effect with lower cell viability of 48% at 100 ng/nL and 29% at 500 ng/mL compared to free drug and PMMSN, along with an IC_50_ value of 65 ng/mL. Similarly, flow cytometry and fluorescence assays showed a higher percentage of apoptotic cells, i.e., around 39% increased expression of apoptotic protein Bax and Caspase-3, and greater Bax/Bcl-2 ratio in the PMMSN-PTX, confirming anti-glioma therapy. In vivo studies showed that tumor growth was significantly inhibited, with lower level of Ki-67 expression and increased tissue necrosis and no significant changes were observed in the other organs tissue section among the different groups, indicating its biosafety (Liu et al. 2024).

Ogun Turan et al., created Fe@MSN, a new formulation comprising of an iron oxide core and a mesoporous silica shell for the delivery of the BTIC-specific inhibitor, 1400W. The major goal of this research was to solve the restriction of insufficient tumor tissue penetration. A two-step medication delivery strategy was created to accomplish this. In the first phase, fibronectin was used as the targeting moiety since it is substantially overexpressed in the extracellular spaces of near-perivascular areas of GBM, where BTICs are mostly found. The fibronectin-targeted Fe@MSN were intended to concentrate preferentially in the vascular and near-perivascular areas of GBM. Following intravenous delivery, these NPs accumulated rapidly within brain tumors, achieving maximal concentration within 3 h. Histological research revealed the exact deposition of the NPs in near-perivascular areas of GBM cells, with no detectable presence in healthy brain tissue. Although excellent nanoparticle deposition was accomplished, their capacity to penetrate deep into the tumor parenchyma was restricted, limiting their anticancer effects. The second phase included applying a moderate, low-power radio-frequency (RF) field frequency that could penetrate whole brain in order to get around this restriction. The NPs’ releasing mechanism was based on the peculiar structure of Fe@MSN particles. The low-power RF field induced fast drug release via mechanical vibration than heating. The iron oxide cores within NPs aligned with the applied RF field by overcoming viscosity and thermal forces when it was exposed to an alternating magnetic field. The alignment process was driven by Néel and Brownian relaxation processes, with the latter being the primary mechanism for the Fe@MSN. The RF field’s vibration provided kinetic energy which released drug trapped within mesoporous silica shell and allow them to diffuse throughout the tumor tissue. In vivo, experiments using BTIC-derived GBM xenografts demonstrated the efficacy of this treatment technique by inhibiting tumor growth and increasing survival rates. This case study illustrates the potential of multicomponent NPs, ligand targeting strategies, and RF-triggered drug release to address GBM treatment challenges and disrupt BTIC populations (Turan et al. 2019).

Ultrasmall large pore silica nanoparticles (USLP) have been shown in a study by Janjua et al., to have potential as a DDS in GBM therapy. According to the study’s findings, drugs may be delivered via USLP-based DDS to the GBM tissue and over the BBB. In a 3D spheroidal model of GBM, the PEGylated nanosystem with Lf as the targeting agent was shown to have excellent penetrating ability and to have penetrated BBB in vitro. The in vitro BBB model demonstrated that endothelial cells in brain rapidly absorbed NPs through transcytosis, which was facilitated due to their interaction at the tight junction proteins. Furthermore, even at low doses, a fluorescent signal was seen in the monolayer of hCMEC/D3 cells, indicating effective nanoparticle uptake in the model. The fluorescent signal increased at higher concentrations, suggesting concentration-dependent uptake of the NPs. The in vitro results indicate that created formulation decreased TMZ efflux from BBB and increased TMZ’s cytotoxic activity against GBM cells (i.e., U87 and GL261). GBM induces resistance by inventing pathways for TMZ efflux. Thus, this approach appears as a potential candidate. Similarly, the in vivo study, depicted the potential of a USLP-based DDS to accumulate in the brain, which indicated the permeability to BBB. Also, Lf coating appeared to have accelerated the delivery time to brain tissue. This was indicated by the fluorescent signal peaking at 1 h compared with NPs without Lf coating (Janjua et al. 2023).

#### Gene therapy

MSNs have surfaced as highly promising carrier for gene delivery, due to their substantial surface area, ample pore size, and adjustable features that can safeguard genetic material against deterioration. Several inquiries have confirmed the efficacy of MSNs as gene carriers by achieving high transfection efficiency and low cytotoxicity.

Huaijun Fei et al., developed TMZ loaded MSN capped by Gint4.1-siHDGF chimera for their co-delivery for GBM therapy. The in vitro assessment demonstrated that gene enables controlled release of TMZ from the nanosystem, with almost 82% drug released over 56 h at pH 5.0. Sequential gene and drug administration at 48 h intervals substantially improved anti-GBM effects with U87 cell survival significantly reduced. Moreover, BBB traversal and GBM cell uptake study showed that these conjugated nanoparticles depicted improved BBB penetration and GBM cell uptake, with higher fluorescence intensities in U87 cells and deep penetration in 3D GBM spheroid model. Blocking of scavenger receptor (SR) decreased fluorescence signals, depicting SR-dependent BBB traversal and effective targeting of PDGFR-β on GBM cells. The in vitro inhibition effect on U87 cells was found to downregulate HDGF expression in U87 cells, leading to a 38.9% cell apoptosis rate and strongly inhibited GBM cell viability, migration, and invasion. Similarly, RNA sequencing revealed involvement of PI3K-AKT and p53 pathways in anti-GBM effects. The in vivo targeting study displayed enhanced GBM targeting and prolonged in vivo fluorescence, with higher fluorescence in GBM-bearing brains at 24 h post-injection compared to other groups. The C6-conjugated nanoparticles resulted in strong fluorescence specifically in the GBM regions, suggesting an effective accumulation and GBM-specific binding. In vivo anti-tumor and safety evaluation showed that TMSN@sHG inhibited GBM growth in U87-Luc xenografts, with the smallest tumor size and a median survival time of 58 days compared to 41 days for TMSN@sH and 34 days for free TMZ with MSN@sH. It was also observed that there no significant myelosuppressive effects or damage to liver or kidney function, indicating its effectiveness as an anti-GBM therapy and safety (Fei et al. 2024).

#### Immunotherapy

MSNs have arisen as potential candidates for immunotherapy because of their ability to induce immune responses and deliver immunotherapeutic agents. The loading capacity of the drug and the large surface area of MSNs make them an efficient carrier for immunotherapeutic agents. CpG-ODNs are synthetic DNA molecules that contain unmethylated CpG motifs, activating the immune system by compelling Toll-like receptor 9 (TLR9) on immune cells like dendritic cells (DCs). Ong and colleagues utilized large porous MSNs that were adorned with diminutive GNPs to transport CpG-ODNs into DCs, leading to an elevated expression of co-stimulatory molecules with augmented pro-inflammatory cytokines secretion(Ong et al. 2019). CpG pertains a precise DNA sequence that encompasses a cytosine nucleotide followed by a guanine nucleotide joined by a phosphate molecule, which can trigger the immune system. Synthetic CpG-ODNs have potential therapeutic applications, such as in cancer immunotherapy and vaccine adjuvants (Jahrsdörfer and Weiner 2008). Lee and colleagues (2020) utilized hollow MSNs with large pores for ovalbumin (OVA) delivery to DCs, activating an antigen-specific immune response. The nano vaccines led to a rise of antigen-specific CD8 + T cells population, resulting in reduced tumor growth and improved survival in mice. PEI refers to polyethyleneimine, a polymer coating used to enhance the stability and delivery efficiency of MSNs(Yuba et al. 2014). OVA is a model antigen commonly used in immunology research. DCs, or dendritic cells, are integral immune cells involved in the pivotal task of initiating and regulating immune responses. CD8 + T cells are a subdivision of immune cells that are involved in killing infected or cancerous cells (Lee et al. 2020).

#### Photodynamic therapy

MSNs, which have mesoscale pores, are showing great potential for cancer treatment through photodynamic therapy (PDT). PDT is a non-invasive therapy that uses light-sensitive chemicals to generate reactive oxygen species (ROS) that selectively target cancer cells. MSNs are particularly suitable for PDT due to their unique characteristics. Their large surface area and pore volume enable high drug loading and effective delivery of photosensitizers (PSs) to cancer cells. Additionally, their mesoporous structure allows for regulated release of PSs, only activating when necessary, while functionalization with targeting ligands can improve therapy specificity. This targeted administration minimizes the negative effects of chemotherapy. MSNs have also been found to have low toxicity and are biocompatible, making them a suitable option for human application (Bayir et al. 2018; Parra et al. 2021).

Pedrozo da Silva et al. have engineered a specialized nanoplatform for cancer photodynamic therapy comprising silica nanoparticles (SiNPs) functionalized with Erythrosine B, an established photosensitizer. Amino-functionalized SiNPs were synthesized and subsequently conjugated with biotin to evaluate the effect of surface modification on therapeutic performance. The degree of biotin functionalization reached 4.50 × 10⁻⁶ mol g⁻^1^ (equivalent to 1.10 mg g⁻^1^) on biotinylated SiNP-B. Loading efficiency of Erythrosine B was markedly higher in non-biotinylated SiNPs (87.4 ± 1.2%) compared to biotinylated SiNP-B (41.7 ± 4.0%), reflecting alterations in surface properties post-functionalization. Adsorption kinetics were best described by a bi-exponential first-order model, with initial rate constants (k₁) of 0.12 min⁻^1^ for SiNP and 0.83 min⁻^1^ for SiNP-B. Furthermore, the Langmuir model provided an optimal fit for the SiNP adsorption data, revealing a maximum capacity (qmax) of 206.68 mg g⁻^1^. In contrast, SiNP-B conformed to the Freundlich model, with a KF value of 19.94 mg g⁻^1^. Controlled release studies demonstrated that Erythrosine B release from SiNP-B was minimal (~ 3% after 24 h), with an associated release rate constant of 0.013 min⁻^1^. Photodynamic treatment using both ERY/SiNP and ERY/SiNP-B resulted in a substantial cytotoxic effect, leading to an approximate 50% reduction in T98G glioblastoma cell viability. The efficient adsorption of Erythrosine B can be attributed to favorable interactions with amino groups present on the surfaces of NPs. Notably, biotin functionalization resulted in a decrease in specific surface area and partial disruption of the pore architecture. Both silica nanoparticle formulations exhibited pronounced singlet oxygen generation and effective inhibition of glioblastoma cell growth. These findings suggest that biotin-functionalized silica nanoparticles are a promising platform for targeted photodynamic therapy against GBM.

In a study conducted by Zhu et al., they developed a mesoporous ruthenium nano-system (MRN) that had dual targeting functionality by using Tf and aptamer AS1411 (Apt) that were grafted onto the surface of the MRN. The MRN had a high loading capacity and was conjugated with Tf/Apt via redox-cleavable disulfide bonds that served as a capping agent as well as a targeting ligand. The goal of this design was to allow for efficient BBB penetration and selective targeting of glioma cells. Both in vitro and in vivo, the created MRN nano-system selectively eliminated glioma cells. The released anti-tumor chemical promoted death in glioma cells via singlet oxygen production during PDT. The combined application of chemical PDT with the targeted nano-system showed synergistic results in treating brain tumors, implying that the MRN nano-system, with its dual targeting function and PDT capabilities, offers potential as a synergistic method for brain tumor therapy (Zhu et al. 2018).

Another study by Kang et al., discussed the development of nanoformulation SIWV-pSiNP(ICG) for targeted photothermal therapy of GBM. This nanoformulation, which consists of ICG (Indocyanine green), showed an increased photodynamic effect under NIR light irradiation. That is, it generated more ROS than free ICG, indicating its superiority. Moreover, the nanoformulation also exhibited non-toxicity and biocompatibility in U87MG cells and the Huh7 cell line. It was further confirmed that the GBM homing ability of the developed nanoformulation via cellular uptake and internalization study signified its specificity to the U87MG cells, which overexpress the caveolin receptor. The photodynamic properties of the developed nanoformulation were also analyzed. The data obtained from the study showed that significant cytotoxicity was observed for the developed nanoformulation-treated GBM cells with laser irradiation, higher ablation ratio was observed when compared with the free Icg. This nanoparticle-based approach has been explored to overcome the limitations. The study concluded that the developed nanoformulation exhibited superior therapeutic efficacy and excellent biocompatibility in GBM xenograft mice, paving the way for a novel and efficient strategy to enhance ODT efficacy for targeted GBM therapy (Kang et al. 2022).

#### Photothermal therapy

In recent years, researchers have shown great interest in MSNs as a potential treatment option for cancer through photothermal therapy (PTT). This therapy involves the use of NPs that can absorb near-infrared (NIR) light and convert it into heat, which results in localized hyperthermia that can destroy cancer cells. MSNs offer several advantages in this context, such as their high surface area and pore volume, which facilitate the efficient loading and delivery of photothermal agents to cancer cells. As such, MSNs present considerable potential as a novel and efficacious avenue for treating cancer. Additionally, these agents can be customized with targeting ligands such as antibodies or peptides, thereby augmenting treatment specificity while concurrently mitigating the risk of any undesirable side effects (Argyo et al. 2014; Shah et al. 2022).

MSNs are highly compatible with human biology and possess a low toxicity profile, rendering them ideal for use in human applications. PTT using MSNs presents a promising avenue for non-invasive cancer treatment. By selectively targeting cancer cells while minimizing damage to healthy tissues, MSN-based PTT has the potential to be a highly effective treatment option. To summarize, MSNs have shown promise in cancer treatment using PTT due to their distinctive features, such as targeted delivery, high drug loading, good biocompatibility, and low toxicity. While more investigation is necessary to uncover the complete potential of MSNs in PTT for cancer treatment, the initial outcomes are optimistic(Pasqua et al. 2016; Gao et al. 2020).

In a study conducted by Li et al*.,* the authors developed an RGD peptide-modified erythrocyte membrane MSN-loaded miR-137 for the theranostic application in glioblastomas. The study showed that the developed nanoparticles exhibited excellent biocompatibility, with a hemolysis rate of < 5% whereas cell viability was > 90% at higher concentrations, along with reduction in phagocytosis due to coating and enhanced tumor targeting through RGD modification depicted by the highest fluorescence intensity in RGD modified nanoparticles when compared to the non-targeted ones. The in vitro cell line assessment of the nanoparticles in the U87 cells showed a strong anti-proliferative effect with only 10% cell survival and around threefold increase in miR-137 levels. Furthermore, in vivo studies depicted that significant NIR imaging signals and temperature increase (~ 13℃) at the site of the tumor upon laser irradiation. NIR-fluorescence imaging confirmed a better tumor targeting efficiency with the RGD-modified nanoparticles with high imaging potency. It was also found that tumor inhibition rates in U87 glioblastoma xenograft model were at their highest, at 94.9%, with minimal body fluctuations and fewer side effects (Li et al. 2022a).

#### Sonodynamic therapy

MSNs have emerged as a promising candidate for use in sonodynamic therapy (SDT) for cancer treatment. SDT involves using ultrasound waves and a photosensitizer to activate ROS that can cause cancer cell death. MSNs have been extensively studied for their potential as a carrier of photosensitizers in SDT. They can effectively load and deliver photosensitizers to cancer cells, increasing the effectiveness of the therapy. The high surface area and pore volume of MSNs allow for the efficient delivery of a large amount of photosensitizer, which results in a higher degree of ROS generation upon activation (Vallet-Regí et al. 2018; Jafari et al. 2019). MSNs can also be functionalized with targeting ligands, such as folic acid or antibodies, which can increase the specificity of the treatment by binding to specific receptors on the surface of cancer cells. When exposed to ultrasound waves, the MSNs generate ROS, which causes cell death in cancer cells. MSNs are also biocompatible and have low toxicity, making them suitable for use in humans (Li et al. 2019; Taleghani et al. 2021).

The ability to deliver photosensitizers to specific cancer cells with high efficiency, low toxicity and the potential for targeted delivery make MSNs an attractive option for SDT in cancer treatment. However, further research is needed to fully explore their potential in this area. In conclusion, MSNs have shown great promise as a carrier of photosensitizers in SDT for cancer treatment. Their high surface area and pore volume, biocompatibility, and potential for functionalization with targeting ligands make them an attractive candidate for further research in this field (Živojević et al. 2021; Xu et al. 2022).

### Diagnostic applications

The unique features of MSNs, such as their adjustable size and surface properties, make them highly promising for use in imaging applications. For the diagnosis of the GBM, neuroimaging plays a vital role. Neuroimaging diagnostic techniques include PET, SPECT, CT, MRI, NIR, and FITC labeling, generally performed for the diagnosis, prognosis, and monitoring of the assessment between the pre- and post-treatment.

#### Single Photon Emission Computed Tomography (SPECT)

SPECT is a nuclear medicine technique used as a tool for the differentiation between active tumors and changes in tumors related to the treatment. SPECT and Computed Tomography (CT) are generally used in combination for increased specificity which is due to the precise localization and characterization of the functional findings(Hanif et al. 2017; Dogra et al. 2018). According to a recent study conducted by Dogra et al.,, the clearance and distribution of MSNs were examined in healthy rats. The study focused on investigating how the particle size, surface chemistry, and delivery method affected their performance. The researchers used SPECT/CT to determine the disposition kinetics of MSNs in ten regions of interest. Additionally, it was found that larger MSNs tended to accumulate in the spleen and liver. Moreover, MSNs that featured unshielded amine groups exhibited a reduced rate of circulation when compared to MSNs that had shielded amine groups or MSNs that were neutral in size. The study also found that MSN size was strongly correlated with an important pharmacokinetic parameter, AUC 0–24 h, which defines the nanoparticle concentration over time The study found that smaller-sized MSNs (Mesoporous Silica Nanoparticles) led to a greater number of particles in circulation. Additionally, MSNs with a positive charge were eliminated more quickly but had a higher tendency to accumulate in the liver and spleen. This research highlights the potential of MSNs for imaging applications and brings to light the influence of size and surface properties on their performance in vivo (Israel et al. 2019a, b).

Cheng and his colleagues conducted a study that introduced a new method for tracking neural stem cells (NSCs) in the brain using MSNs facilitated by SPECT imaging. The MSNs were conjugated with 111In, taking advantage of their unique porous feature and large surface area, which provided a stable and non-toxic labeling platform for NSCs. SPECT imaging successfully visualized the migration of NSCs towards glioma xenografts in real-time after intracranial or systemic administration. Mice were imaged using SPECT/CT and optical imaging at different time points. The SPECT signal quantification was conducted using ImageJ software, with regions of interest drawn in the left hemisphere of the brain and the corresponding region on the contralateral side. The positive results indicate the potential for non-invasive multimodal tracking of therapeutic NSCs in brain malignancies using integrated SPECT imaging (Cheng et al. 2016).

The study Gao and his colleagues presents technetium-99 labeled manganese oxide-based mesoporous silica nanoparticles (99mTc-Mn-MSNs) designed for dual-modality imaging using single photon emission computed tomography-magnetic resonance imaging (SPECT-MRI) in cancer diagnosis. Synthesized as PEG-Mn-MSNs, these NPs showcased promising capabilities in multimodal imaging, anti-cancer drug delivery, and therapeutic monitoring. The stepwise modification process yielded stable, well-dispersed NPs, as confirmed by structural characterizations including TEM, STEM, and DLS measurements indicating size increments with surface modifications. Notably, PEG-Mn-MSNs exhibited a remarkable pH-responsive behavior, significantly enhancing T1 relaxivity in acidic environments, promising accurate tumor detection via MRI. In both in vitro as well as in vivo settings, these NPs portrayed pronounced contrast enhancement within tumor tissues, suggesting their potential for precise tumor localization and monitoring. Successful radiochemical labeling, high stability, and preferential accumulation within the tumor were validated through SPECT imaging, emphasizing the applicability of 99mTc-Mn-MSNs-PEG in efficient tumor detection. Further, efficient drug loading, sustained and pH-responsive drug release, along with low in vivo cytotoxicity, underlined the potential clinical utility of these multifunctional nanoprobes in cancer diagnostics and targeted therapy, marking a significant step toward enhanced cancer management strategies (Gao et al. 2016).

#### Magnetic Resonance Imaging (MRI)

MRI is used as a primary diagnosis of GBM. It is a crucial tool for the identification of tumor growth locations with accuracy and the collection of detailed imaging data. This also aids the prediction of patient survival rates with precision, making it an essential resource for healthcare professionals. In their research, Zhongtao Li et. al and their team developed a new MRI contrast agent, called Gd2O3@MSN, using a simple one-step method that increases T1 contrast capacity. This method is not just economical, but also has the capacity to enhance medical diagnosis and treatment by creating innovative and efficient MRI contrast agents. Its potential to revolutionize the field cannot be overstated. T1 contrast refers to the time it takes for protons in tissue to recover their magnetization after being stimulated by a radiofrequency pulse. In simpler terms, it reflects how quickly protons emit signals back to the MRI machine, and T1 contrast capacity indicates the contrast agent’s ability to enhance T1 contrast, resulting in brighter and clearer images. The researchers evaluated the toxicity of Gd2O3@MSN on three types of cells, namely AsPC-1, PaCa-2, and 4T1, using the CCK-8 assay. The results showed that the NPs did not cause any significant harm to the cells after 24 h of exposure, indicating their safety. Moreover, they tested the safety of the contrast agent in healthy rats by injecting them with a high dose of the solution and observing them for a week. None of the rats exhibited observable side effects or toxicity, and all of them survived. The researchers also examined the rats’ main organs and blood tests and found no significant differences, confirming that Gd2O3@MSN is safe for use in living systems. Thus, the study concludes that this new MRI contrast agent has the potential to advance medical diagnosis and treatment, and it is safe for use in living organisms(Shao et al. 2011; Li et al. 2022b).

Hybrid mesoporous composite nanocapsules (HMCNs) have been developed for cancer theranostic and diagnosis by researchers. These nanocapsules utilize nano-biotechnology to respond to and detect the acidic microenvironment of tumors, and they can be used for both MRI and ultrasonography. The mesopores within the shells provide multiple functions such as nanoreactors and nanoreservoirs for the storage and dispersion of manganese oxide (MnOx) NPs. The presence of these confined MnOx NPs which allows for pH-responsive dynamic MRI capabilities. This capability is demonstrated by evaluating the relaxivity rate (r1 values) in the buffer solutions with different pH values. The colour of the HMCNs buffer solution changes gradually in low pH solution (pH 5.0), indicating the dissolution of MnOx NPs. A significantly increased relaxation rate (r1) of the manganese-based mesoporous MRI-T1contrast agents is observed. The r1 value of these agents reaches 8.81 mM^−1^ s^−1^, which is 11 times higher than the neutral condition and almost twice as high as commercially available Gd^III^-based complex agents. This represents the highest r1 value reported for manganese oxide NPs -based MRI-T1 contrast agents. The corresponding decrease in T1 and T2 relaxation times of hydrogen protons in the buffer solution is observed under different Mn concentrations, with more substantial changes in the low pH solution. The relaxivity rates of HMCNs in the low pH solution show a significant increase compared to the high pH solution, making them suitable for pH-responsive MRI. Therefore, the HMCNs offer a versatile platform for dual-modality cancer imaging using MRI and ultrasonography, as well as targeted drug delivery for overcoming drug resistance in tumorous cells (Chen et al. 2012).

Mesoporous silica-coated superparamagnetic manganese ferrite (M-MSN) nanoparticles have been engineered for MRI-based cancer diagnosis and targeted drug delivery. Loaded with the anticancer drug Doxorubicin, these M-MSN were extensively evaluated for their effectiveness in cancer treatment via in vitro cytotoxicity assays, fluorescence microscopy, and apoptosis studies. It was demonstrated that the folate-conjugated NPs (FA-MSN) act as superior MRI contrast agents, showing enhanced T2-weighted MRI contrast towards cancer cells compared to those without folate conjugation. The integration of MSN with MRI technology is a significant development in cancer diagnosis. This technology offers a multifunctional platform that combines a magnetic core for imaging, a porous shell for drug delivery, cancer-specific targeting moieties, and fluorescent molecules for precise imaging. The combination of these features suggests promising biomedical applications (Sahoo et al. 2014).

Despite advancements in GBM treatment, effective therapeutic delivery and early therapy efficacy remain elusive. Significant progress in image-guided and targeted therapy for GBM is essential to improve patient outcomes. This study explores a novel method for non-invasive monitoring of cargo molecule release from theranostic nanoparticles via MRI. The approach involves quantifying changes in longitudinal relaxation time (T1) before and after the pH-responsive release of contrast agents from MSNs targeted to GBM. MSNs were loaded with either the anticancer drug paclitaxel (PTX) or the FDA-approved contrast agent Gadobutrol. The retention of these agents within the MSNs’ pores was ensured by a pH-cleavable hydrazone linkage. In vitro studies using GBM cell lines showed effective delivery of the therapeutic cargo, which was enhanced by further functionalizing MSNs with glioma-homing oligopeptide chlorotoxin (CTX). MRI measurements successfully demonstrated changes in T1 in response to the release of Gadobutrol from the MSNs, highlighting the potential for MRI-based tracking of therapeutic release in tumors. This method could lead to more personalized cancer treatment by enabling real-time monitoring of drug delivery and release in GBM tissues. Additionally, MRI measurements at different pH levels showed significant differences in T1 values, correlating with the enhanced release of Gadobutrol in acidic conditions, typical of the tumor microenvironment. These findings suggest that MRI tracking of contrast agent release from nanotheranostic devices could improve the clinical management of GBM by providing real-time insights into therapeutic delivery and efficacy (Mundžić et al. 2023).

#### Positron Emission Tomography (PET)

PET is a extremely effective tool in the field of oncology due to its exceptional molar concentration resolution, which ranges from 10 − 11 to 10 − 12 mol/L. Additionally, it provides valuable functional information and boasts impressive quantitative capabilities (Abrantes et al. 2020). In PET scanners, annihilation photons interact at opposite sites on the detector ring, forming coincidences that define a line for localization and image reconstruction (Shukla and Kumar 2006).

Radiolabeled MSN, using various isotopes like 64Cu, 89Zr, and 124I, have facilitated highly sensitive and precise PET imaging for tracking organ/tissue distribution and targeting tumors in vivo. Integrating these NPs with PET has enabled the detection of specific lesions, such as integrin-expressing lesions in metastatic melanoma, and the visualization of tumor vasculature. Furthermore, MSN-based PET-guided drug delivery strategies have shown enhanced efficacy in targeting and delivering drugs to tumor sites, improving cancer diagnosis and prognostics. The combination of MSN with PET imaging techniques holds significant promise for advancing cancer diagnosis, enabling early detection, and optimizing therapeutic interventions (Ni et al. 2018).

Cheng Xu and his colleagues conducted a study to create mesoporous silica-coated gold nanorods that have a larger surface area and pore size. These nanorods are ideal for use as both contrast and therapeutic agents for cancer therapy. They discovered that when exposed to an 808 nm NIR laser at a power density of 0.25 W/cm2, the temperature of the solution increased to over 45 °C within 10 min. Additionally, they found that the efficiency of loading DOX onto bGNR@MSN was dependent on the pH of the solution. Scientists harnessed the power of bGNR@MSN’s photothermal effects to accelerate the release of DOX, which led to an uptick in the overall amount of DOX released. They utilized PET imaging to demonstrate that bGNR@MSN(DOX)-PEG effectively zeroed in on the tumor location with a substantial buildup of the drug. PA imaging also verified the heightened concentration of bGNR@MSN(DOX)-PEG at the tumor site. Treatment with bGNR@MSN(DOX)-PEG while being exposed to NIR laser radiation yielded significantly better therapeutic outcomes when compared to individual chemotherapy or photothermal therapy. These findings have considerable potential for revolutionizing cancer detection and treatment in the future(Xu et al. 2018; Fernández-Lodeiro et al. 2021).

The objective of the study done by Chen and his colleagues was to assess the stability of MSNs and porous silicon NPs (dSiO_2_) labelled with ^89^Zr for long-term monitoring through PET imaging. The researchers utilized PET imaging to monitor the stability and biodistribution of ^89^Zr-labeled NPs in vivo. The researchers administered ^89^Zr-labeled MSN and stable silica NPs (dSiO_2_) to mice to monitor the absorption and removal of these NPs over a period of three weeks. The researchers initiated their investigation by administering ^89^Zr-labeled MSNs and porous silicon NPs (dSiO_2_) into mice, monitoring the distribution within their bodies for a span of 3 weeks, approximately encompassing 7 half-lives of ^89^Zr. The utilization of PET imaging and maximum intensity projection images effectively confirmed the high uptake of free ^89^Zr-oxalate in the bones, thereby validating the approach**.** Primary objective of study was to assess the long-term stability and monitoring capabilities of ^89^Zr-labeled MSN and dSiO_2_ through PET imaging. The researchers employed PET imaging to examine the stability and distribution of ^89^Zr-labeled NPs within live mice. The results revealed that mice injected with ^89^Zr-labelled dSiO_2_ displayed a significant increase in bone uptake, starting from the first day post-injection, indicating the release of the free ^89^Zr ions from surface of dSiO_2_. Subsequently, no further increase in bone uptake was observed, suggesting strong binding of the remaining ^89^Zr ions to dSiO_2_ surface. Conversely, mice injected with ^89^Zr-labelled MSN exhibited remarkable in vivo stability throughout the 3-week study period, with minimal bone uptake. This finding indicates that the meso-channels within MSN play a crucial role in safeguarding ^89^Zr from interaction with endogenous chelators. Furthermore, the detachment rate of the ^89^Zr from MSN was observed to be more than 20 times slower than that from dSiO_2_, underscoring the criticality of the meso-channels in preserving the stability of ^89^Zr-labeled NPs (Chen et al. 2015).

#### X-ray

Combining radiotherapy with immune checkpoint blockade therapy offers a promising approach to treating glioblastoma multiforme, yet challenges such as limited effectiveness and immune-related adverse events (irAEs) persist due to the failure in targeting immunomodulators directly to the tumor microenvironment. To address this, we developed a biomimetic nanoplatform that combines a genetically modified mesenchymal stem cell (MSC) membrane with a bioactive nanoparticle core for chemokine-directed radioimmunotherapy of GBM. The CCR2-overexpressing MSC membrane achieves radiation-induced tropism toward the abundant chemokine ligand CCL2 in irradiated gliomas. The nanoparticle core, comprising diselenide-bridged mesoporous silica nanoparticles and PD-L1 antibodies (αPD-L1), enables X-ray-responsive drug release and radiosensitization. In two murine models with orthotopic GBM tumors, this nano platform reinvigorated immunogenic cell death and augmented the efficacy and specificity of GBM radioimmunotherapy with reduced irAEs. We found remarkably elevated secretion of CCL2 in irradiated tumors, which recruits MSCs via CCR2 on their membranes. CCR2-SCM@MSN nanoparticles display enhanced accumulation and prolonged retention in irradiated GBM tissues due to the CCR2-CCL2 axis. X-ray irradiation promotes the degradation of the diselenide-bridged MSN framework, facilitating αPD-L1 release within the tumor, which limits irAEs in healthy tissues. Additionally, the nanoplatform’s radiosensitization properties enhance PD-L1 expression, further blocking the PD-1/PD-L1 axis, which neutralizes RT-induced immunosuppression. This innovative strategy ensures the spatial synchronization of RT with immunotherapy, enhancing the local RT efficacy and immunogenic cell death effect, and potentially improving synergy with other therapeutic approaches (Wang et al. 2024).

Radiotherapy-resistant glioblastoma (rrGBM) remains a significant clinical challenge due to its high infiltrative growth and resistance to standard treatments. To address this, we developed a biodegradable selenium-engineered mesoporous silica nanocapsule (SeMSN) designed for targeted RNA interference (RNAi) and activated by low-dose X-ray irradiation. These nanocapsules show excellent physiological stability, efficient blood–brain barrier transcytosis, and notable rrGBM accumulation. Upon X-ray exposure, SeMSNs release cofilin-1 siRNA, effectively knocking down its expression and inhibiting tumor invasion. The X-ray irradiation exacerbates hypoxia within the tumor environment, triggering the conversion of metronidazole polymers into electron-affinity aminoimidazole products, which further enhance the radiosensitization effect. This process stabilizes reactive oxygen species (ROS)-induced DNA damage, preventing repair and sensitizing the tumor cells to apoptosis. In vivo studies demonstrated that SeMSN(siCFL1)@P(MNs)Ang2 significantly reduces tumor growth and invasion, prolongs survival, and exhibits minimal toxicity. This innovative approach highlights the synergistic relationship between X-ray irradiation and mesoporous silica nanocapsules, presenting a promising radiosensitizer that could substantially improve therapeutic outcomes for rrGBM patients with low-dose X-ray treatment (Tang et al. 2023) Table 2.

**Table 2:** Imaging Techniques with specifications, advantages, and disadvantages

Imaging techniques	Imaging source	Spatial resolution	Tissue penetration depth	Sensitivity	Agents	Advantage	Disadvantage	References
SPECT	Gama ray	About 6–7 mm	Limitless	High	Radionuclides	Viewing of the reconstructed image in multiple planes Ability to separate overlapping images	Exposure to minute amounts of radiation Lack of accessibility	(Israel et al. 2019a, b; Forte et al. 2020)
CT	X-ray	50–200 µm	Limitless	Not specified	High-atomic-number atoms	Fast and accurate Non-invasive and painless	Radiation exposure Expensive Not suitable for medical conditions like pregnancy and renal disorders	(Griffeth 2005; Lusic and Grinstaff 2013)
MRI	Radio wave	25–100 µm	Limitless	Low	Para-(Gd^3+^)/ superparamagnetic (Fe_3_O_4_) materials	Non-invasive and painless Does not use Radiation Better differentiation of soft tissue	Expensive Not fit for patients with Claustrophobia, implants, etc More time-consuming than the rest	(Xiao et al. 2016)
PET	Gama ray	About 1–2 mm	Limitless	High	Radionuclides	Detects distant metastatic lesions faster than other techniques Functional and anatomical information in single testing	Limited spatial resolution Inter-observer variability observed	(Abrantes et al. 2020; Jadvar 2022)
Optical fluorescence imaging	Visible or NIR	2–3 mm	< 1 cm	Medium	Fluorescent dyes – FITC,	Used to identify the margins of the tumors	Limited by the opacity of the human body	(Kampschulte et al. 2016; Kalimuthu et al. 2017; Teng et al. 2020; Schouw et al. 2021)

#### Optical fluorescence imaging

MSNs have high loading capacity, stability, and adjustable pore size. They are easy to functionalize due to surface characteristics. In cancer treatment, MSN-based nanoprobes have significantly enhanced fluorescence imaging by improving solubility, preventing aggregation, increasing quantum yield, ensuring chemical stability, and resisting photobleaching of contrast agents. Moreover, MSNs have bolstered photoacoustic imaging by stabilizing nanostructures and enhancing imaging signals, showcasing potential applications in cancer diagnosis and therapy. However, challenges persist in ensuring reproducibility during synthesis and addressing long-term safety concerns related to non-degradable contrast agents. Overcoming these hurdles could pave the way for clinical translation, offering promising avenues for improved cancer diagnostics, targeted therapy, and treatment monitoring through advanced optical imaging techniques (Sun et al. 2021).

Fluorescence-guided surgery (FGS) targeted towards tumors has become a highly promising method for visualizing solid tumors during operations. FGS utilizes the Stokes shift principle, where the energy difference between absorption and emission spectra of a fluorescent contrast agent enables precise surgical guidance (Mieog et al. 2021). In another study, Heidegger and colleagues aimed to track specific immune cells using MSNs conjugated with a fluorescent dye. The researchers added amine groups to the MSNs and then combined them with a fluorescent dye known as fluorescein isothiocyanate (FITC) to enhance their colloidal stability. They then incubated splenocytes isolated from mice with the FITC-labeled MSNs and observed that the immune cells took up the MSNs in a concentration-dependent manner. This indicated that the synthesized fluorescent dye-conjugated MSNs can potentially be used as a tool to trace specific immune cells in vivo (Nakamura et al. 2015; Heidegger et al. 2016).

Functionalized fluorescent silica nanoparticles, incorporating rhodamine 101 (R101) dye, were engineered to selectively target cancer cells for imaging analysis. Optimization techniques increased dye brightness within the NPs, which is essential for sensitive imaging. Surface modifications with PEG and folic acid enhanced stability in water and improved specificity to tumourous cells expressing folate receptors. In vitro assays revealed effective internalization of these NPs into cancer cells’ lysosomes, illustrating their potential for precise cancer imaging and diagnostics. Despite some stability and cytotoxicity challenges, these engineered MSNs demonstrate significant potential for Optical Fluorescence Imaging in cancer treatment (Prieto-Montero et al. 2020).

## Toxicity aspects

The potential harm that MSNs may cause in medical applications has arisen as a major issue in recent years. As the production and use of MSNs has increased, so has the risk of workplace exposure to humans. Exposure to these particles can occur through multiple routes, including inhalation, ingestion, or skin contact. These nanoparticles can accumulate in different organs by circulating in the body, which is why thorough toxicity testing is necessary both in vitro and in vivo (Murugadoss et al. 2017; Mebert et al. 2017).

Hozayen and colleagues investigated the impact of MSNs on the lungs of Wistar rats. For four weeks, rats were injected intraperitoneally with NPs ranging from 25 to 200 mg/kg body weight per day. According to the study, there was an increase in the formation of ROS, MDA, and NO in the lungs of rats, which was dependent on the dose. Specifically, rats that were given 200 mg of NPs per kg of body weight daily showed a significant increase of approximately 2.7 times, 1.9 times, and 2.3 times in the generation of ROS, MDA, and NO, respectively, compared to rats given a daily dose of 25 mg per kg of body weight (Hozayen et al. 2019). Chauhan and their team conducted a study investigating the effects of MSNs on two types of cells: bone marrow mononuclear cells (BM-MNCs) and human neuroblastoma SH-SY5Y cells. The study revealed that low to moderate concentrations of MSNs (5–25 µm) did not cause any harm to BM-MNCs. MSNs, however, caused cytotoxicity after two hours of exposure at greater concentrations (50–100 µm).

Similarly, the study discovered that MSNs were not hazardous to SH-SY5Y cells at concentrations of 1–10 ng/L after 24 h of exposure, while greater quantities (1–100 g/L) were toxic after 48 and 72 h. MSNs’ concentration and surface chemistry are critical factors in their potential brain toxicity. Mahmoud and colleagues investigated the processes behind MSN-induced liver damage in rats. The researchers gave the rats varied dosages of MSNs for 30 days and found that the NPs increased blood levels of ALT and AST, two liver enzymes, in a dose-dependent way. The study also found that as the quantity of NPs increased, the concentrations of ROS, MDA, and NO rose, indicating that the NPs induced oxidative stress and enhanced ROS generation in the liver. Moreover, the study found that MSNs inhibited the activity of key antioxidants, including GSH, SOD, and CAT, as well as the PPAR signaling pathway. This pathway is in charge of controlling the generation of antioxidant enzymes as well as reducing inflammation and fibrosis in the liver(Chauhan et al. 2018).

Put simply, the study demonstrated that MSNs can induce liver damage in rats by elevating liver enzyme levels, triggering oxidative stress, and inhibiting liver antioxidants. Additionally, it revealed that MSNs impede the PPARγ signaling pathway, which is responsible for regulating antioxidant enzymes, preventing inflammation, and averting fibrosis. Despite the general belief that MSNs have minimal toxicity, their biocompatibility and toxicity can be affected by several factors, such as particle size, shape, and surface charge. These determinants significantly impact the overall safety and effectiveness of MSNs. To mitigate the potential toxicity of MSNs, various strategies have been developed, including coating them with biocompatible polymers or utilizing controlled release systems to limit the amount of drug released at once. Lastly, it’s crucial to conduct detailed biocompatibility and toxicity studies to assess the safety of MSNs for their application in the field of biomedicine (Mahmoud et al. 2019; Maccuaig et al. 2022).

## Clinical trials related to MSNs

MSNs have drawn significant interest in the domain of clinical research, especially in the context of drug delivery, targeted therapies, and diagnostics. These MSNs possess many exceptional properties, making them promising candidates for biomedical applications. Clinical trials related to MSNs have explored oral drug delivery, plasmonic resonance, photothermal therapy, diagnostics, and multifunctional biomedical applications. The observations from these trials have yielded significant insights into the clinical use of MSNs, thereby paving the way for their potential incorporation into medical practice. The role of MSNs in improving healthcare and medical research is expected to expand significantly as the number of clinical trials involving these MSNs continues to grow. Table 3 summarizes ongoing and completed clinical trials, highlighting the use of MSNs or silica-based nanoparticles in targeted drug delivery, imaging, and other therapeutic applications, with relevance to cancer therapy, including potential implications for glioblastoma multiforme (GBM) treatment.

**Table 3:** Clinical Trials Involving Mesoporous Silica Nanoparticles (MSNs) and Related Silica-Based Nanomaterials for Cancer and Other Biomedical Applications

S. No	NCT number	Title	Status	Condition	Interventions	Sponsor	Phases
1	NCT02106598	Targeted Silica Nanoparticles for Real-Time Image-Guided Intraoperative Mapping of Nodal Metastases	Recruiting	Head and Neck Melanoma	Drug: fluorescent cRGDY-PEG-Cy5.5-C dots	Memorial Sloan Kettering Cancer Center	Phase 1, Phase 2
2	NCT04167969	Use of Nanoparticles to Guide the Surgical Treatment of Prostate Cancer	Recruiting	Prostate Cancer	Drug: (64Cu)-labelled PSMA-targeting particle tracer or 64Cu-NOTA-PSMAi-PEG-Cy5.5-C' dots Diagnostic Test used: PET/MRI Other: Blood and urine sampling Procedure: laparoscopic radical prostatectomy and bilateral pelvic LN dissection or a salvage lymph node dissection	Memorial Sloan Kettering Cancer Center	Phase 1
3	NCT01160523	To promote Consistent Shoe Use Among Children who are at High Risk for Podoconiosis	Completed	Podoconiosis Non-Filarial Elephantiasis	-	National Human Genome Research Institute (NHGRI)	-
4	NCT01270139	Plasmonic Nanophotothermal Therapy of Atherosclerosis (NANOM-FIM)	Completed	Stable Angina Heart Failure Atherosclerosis Multivessel Coronary Artery Disease	Procedure: Transplantation of nanoparticles Procedure: Transplantation of iron-bearing nanoparticles Device: Stenting	Ural State Medical University	Not Applicable
5	NCT01436123	Plasmonic Photothermal and Stem Cell Therapy of Atherosclerosis Versus Stenting (NANOM PCI)	Terminated (The study was terminated under the political pressure of the Federal Security Service of the Russian Federation (FSB) and the Russian Society of Cardiology)	Coronary Artery Disease Atherosclerosis	Other: Stenting and micro-infusion of NP- Device: Implantation of everolimus-eluting stent	Ural State Medical University	Phase 1
6	NCT02507596	Evaluation of Nano-crystalline Hydroxyapatite Silica Gel in Management of Periodontal Intrabody Defects	Completed	Chronic Periodontitis	Biological: Nano-crystalline hydroxyapatite silica gel Procedure: open flap debridement	Cairo University	Not Applicable

### Challenges in clinical translation of MSNs for GBM therapy

Despite their promising features, such as high drug-loading capacity, tunable pore size, and biocompatibility, MSNs have not advanced to widespread clinical trials for GBM therapy due to significant challenges. Safety concerns persist, as MSNs can induce oxidative stress and organ accumulation, with studies showing dose-dependent increases in reactive oxygen species and liver enzymes in rats. Scalability is another hurdle, as producing MSNs with consistent properties under Good Manufacturing Practices is complex and costly, with variations in synthesis affecting drug release and efficacy. Moreover, the absence of standardized regulatory frameworks for nanoparticle therapeutics complicates their approval, necessitating extensive data on pharmacokinetics and degradation, which are difficult to obtain due to the inorganic nature of MSNs (Abdel-Maksoud et al. 2025). The restrictive human BBB and GBM’s molecular heterogeneity, with varied receptor expression (e.g., EGFR, CD44), necessitate personalized MSN designs, which are logistically difficult to implement. The resistance to TMZ and competition from established therapies, such as tumor-treating fields, further limit the demonstrated clinical efficacy of MSNs.. Moreover, high development costs, long regulatory timelines, and the limited market size for GBM deter commercial investment (Chen et al. 2024). To overcome these barriers, future efforts must focus on long-term toxicity studies, scalable synthesis, clear regulatory guidelines, personalized formulations, and synergistic therapies to realize MSNs’ potential in improving GBM treatment outcomes.

## Concluding remarks and future prospects

Mesoporous silica nanoparticles hold significant promise in the treatment of glioblastoma multiforme, a notably aggressive and lethal form of brain cancer. MSNs are capable of encapsulating drugs and can be functionalized with various molecules on their surface, making them excellent candidates for overcoming the blood–brain barrier, a major obstacle in both the diagnosis and treatment of brain cancer. While concerns about their toxicity exist, further research is essential to assess the safety and efficacy of MSNs in GBM treatment. Recent advancements suggest that MSNs can be engineered to minimize harmful effects, highlighting their potential in clinical applications. Innovative drug delivery systems using MSNs have been developed for GBM therapy. Notably, multicomponent nanoparticles comprising a magnetic core and mesoporous silica shell have shown the ability to navigate across the BBB and penetrate cancer cells, thereby improving drug accumulation within tumor cells and enhancing treatment efficacy. Personalized medication, based on unique cancer characteristics and genetic profiles, represents another promising aspect of MSN-based therapies, potentially leading to more effective and individualized GBM treatments.

Despite significant advancements, several barriers hinder the successful clinical application of MSNs in GBM treatment. Addressing these challenges requires a multifaceted approach that prioritizes safety, effective treatment strategies, and seamless integration into clinical practice. Comprehensive investigations into biocompatibility, biodistribution, immune response, elimination pathways, degradation mechanisms, and overall toxicity of MSNs are crucial. Understanding these factors is vital to ensure the safe and efficient use of MSNs in clinical settings. Tailoring the structural composition of MSNs, surface modifications, and refining drug-loading methods can significantly reduce potential side effects and enhance biological compatibility. Computational tools and predictive models can expedite the design process by anticipating toxicological profiles, facilitating safer MSN development. Overcoming GBM treatment hurdles involves developing MSN-based drug delivery systems capable of targeting diverse tumor cell populations. These systems can be designed to respond to specific biomarkers or environmental cues within the tumor microenvironment, ensuring more precise and effective treatment. To counter drug resistance commonly encountered in GBM treatment, stimuli-responsive drug carriers that release therapeutic agents in response to specific tumor environment triggers are essential. These innovative approaches help bypass resistance mechanisms, ensuring effective delivery of the therapeutic payload.

MSNs can also enhance drug delivery when combined with imaging techniques, extending the circulation period of drugs and improving tumor tissue penetration while carrying imaging agents that aid in accurate diagnosis and treatment monitoring. Exploring synergistic therapies, such as combining MSNs with focused ultrasound therapy or immunotherapy, represents an exciting avenue for improving GBM treatment outcomes. For instance, focused ultrasound can trigger drug delivery from MSNs within tumors, enhancing localized delivery while minimizing systemic exposure. Additionally, leveraging MSNs to deliver immune-stimulatory agents can enhance the body’s immune response against tumor cells, improving immunotherapy effectiveness.

Future research should focus on elucidating the mechanisms of MSN action within the GBM microenvironment through in-depth in vivo studies. Large-scale clinical trials are necessary to evaluate the safety and effectiveness of MSN-based therapies in GBM patients and determine their clinical viability. Clear regulatory frameworks are also crucial to guide MSN development, ensuring patient safety and expediting clinical integration. The future prospects of MSNs in GBM treatment are vast and promising. Advances in nanotechnology could enable the development of multifunctional MSNs capable of delivering multiple therapeutic agents simultaneously, targeting various pathways involved in GBM progression and resistance. Additionally, MSNs could be engineered to respond to external stimuli such as pH, temperature, or light, providing controlled and site-specific drug release. Integration with emerging technologies like CRISPR/Cas9 gene editing could also be explored, allowing for precise genetic modifications within tumor cells to enhance treatment efficacy.

Furthermore, advancements in personalized medicine could see MSNs tailored to individual patient profiles, optimizing therapeutic outcomes and minimizing adverse effects. By incorporating patient-specific data, such as genetic markers and tumor microenvironment characteristics, MSN-based treatments could be customized for maximum efficacy. Collaborative efforts between researchers, clinicians, and regulatory bodies will be essential in translating these innovative approaches from the laboratory to the clinic, ultimately improving survival rates and quality of life for GBM patients. Despite the existing challenges, the potential of MSNs in GBM treatment remains promising. By addressing safety concerns, devising effective treatment strategies, exploring synergistic therapies, and fostering comprehensive research and clinical translation, MSNs stand poised to revolutionize GBM treatment and potentially improve patient outcomes in the future.

## Abbreviations

AASA: Aerosol-assisted self-assembly ​
AFM: Atomic force microscopy ​
AKT: Protein kinase B ​
ATP: Adenosine triphosphate ​
BSA: Bovine serum albumin ​
BTB: Blood-tumor barrier ​
BTIC: Brain tumor-initiating cell
CAR T cells: Chimeric antigen receptor T cells ​
CD44: Cluster of differentiation 44 cell surface receptor ​
CNS: Central nervous system ​
CTX: Chlorotoxin ​
DDS: Drug delivery system ​
DOX: Doxorubicin ​
DTX: Docetaxel ​
DHE: Dihydroethidium
EPR: Enhanced permeability and retention ​
EGFR: Epidermal growth factor receptor ​
EGFRvIII: Variant III of epidermal growth factor receptor ​
FA: Folic acid ​
FDA: Food and Drug Administration ​
FITC: Fluorescein isothiocyanate ​
GBC: Glioblastoma
GBM: Glioblastoma multiforme ​
GSH: Glutathione
GSCs: Glioblastoma stem cells ​
HA: Hyaluronic acid ​
HGF: Hepatocyte growth factor ​
IC50: Half maximal inhibitory concentration ​
IDH: Isocitrate dehydrogenase ​
IL-13: Interleukin-13 ​
IL13R2: Interleukin 13 receptor alpha 2 ​
Lf: Lactoferrin ​
LOH 10q: Loss of heterozygosity on chromosome 10q
LRP1: Low-density lipoprotein receptor-related protein 1 ​
MDA: Malondialdehyde
MAPK: Mitogen-activated protein kinase ​
MGMT: O6-methylguanine-DNA methyl transferase ​
MMPs: Matrix metalloproteinases ​
MSNs: Mesoporous silica nanoparticles ​
NDDS: Novel drug delivery system ​
NIR: Near-infrared ​
NPs: Nanoparticles ​
OVA: Ovalbumin ​
PARP: PolyADP-ribose polymerase ​
PDT: Photodynamic therapy ​
PDGF: Platelet-derived growth factor ​
PD-L1: Programmed death-ligand 1 ​
PET: Positron Emission Tomography ​
PI3K: Phosphatidylinositol 3-kinase ​
PEG: Polyethylene glycol ​
PTT: Photothermal therapy ​
PTEN: Phosphatase and tensin homolog ​
PTX: Paclitaxel ​
RAF: Rapidly accelerated fibrosarcoma ​
RAS: Rat sarcoma ​
ROS: Reactive oxygen species ​
rrGBM: Radiotherapy-resistant glioblastoma ​
SERT: Sensitizer enhancement ratio ​
SEM: Scanning electron microscopy ​
SOD: Superoxide dismutase
SR: Scavenger receptor ​
SDT: Sonodynamic therapy ​
SPECT: Single Photon Emission Computed Tomography ​
TAMs: Tumor-associated macrophages ​
TCGA: The Cancer Genome Atlas Program ​
TEOS: Tetraethyl orthosilicate ​
TEM: Transmission electron microscopy ​
TME: Tumor microenvironment ​
TMZ: Temozolomide ​
TP53: Tumor protein p53 ​
Tf: Transferrin ​
USLP: Ultrasmall large pore silica nanoparticles ​
VEGF: Vascular endothelial growth factor ​
VPA: Valproic acid ​
VM: Vasculogenic mimicry ​
WHO: World Health Organization ​
